# Transitioning from One Electronic Health Record to Another: A Systematic Review

**DOI:** 10.1007/s11606-023-08276-3

**Published:** 2023-10-05

**Authors:** Isomi M Miake-Lye, Alison M. Cogan, Selene Mak, Julian Brunner, Seppo Rinne, Catherine E. Brayton, Ariella Krones, Travis E. Ross, Jason T. Burton, Michael Weiner

**Affiliations:** 1 Center for the Study of Healthcare Innovation, Implementation and Policy (CSHIIP), VA Greater Los Angeles Healthcare System, Los Angeles, CA USA; 2 Department of Health Policy and Management, UCLA Fielding School of Public Health, Los Angeles, CA USA; 3 Mrs. T. H. Chan Division of Occupational Science and Occupational Therapy, Herman Ostrow School of Dentistry, University of Southern California, Los Angeles, CA USA; 4 Center for Healthcare Organization and Implementation Research (CHOIR), VA Bedford Healthcare System, Bedford, MA USA; 5 Department of Medicine, Boston University School of Medicine, Boston, MA USA; 6 Department of Pulmonary and Critical Care Medicine, VA West Roxbury Medical Center, West Roxbury, MA USA; 7 Pain Research, Informatics, Multi-Morbidities, and Education (PRIME) Center, VA West Haven Medical Center, West Haven, CT USA; 8 Yale Center for Medical Informatics, New Haven, CT USA; 9 Louise M. Darling Biomedical Library, University of California Los Angeles, Los Angeles, CA USA; 10 Center for Health Information and Communication, Department of Veterans Affairs, Veterans Health Administration, Health Services Research and Development Service CIN 13-416, Richard L. Roudebush VA Medical Center, IN Indianapolis, USA; 11 Regenstrief Institute, Inc., Indianapolis, IN USA; 12 Department of Medicine, Indiana University School of Medicine, Indianapolis, IN USA

**Keywords:** systematic review, electronic health records, organizational change

## Abstract

**Background:**

Transitioning to a new electronic health record (EHR) presents different challenges than transitions from paper to electronic records. We synthesized the body of peer-reviewed literature on EHR-to-EHR transitions to evaluate the generalizability of published work and identify knowledge gaps where more evidence is needed.

**Methods:**

We conducted a broad search in PubMed through July 2022 and collected all publications from two prior reviews. Peer-reviewed publications reporting on data from an EHR-to-EHR transition were included. We extracted data on study design, setting, sample size, EHR systems involved, dates of transition and data collection, outcomes reported, and key findings.

**Results:**

The 40 included publications were grouped into thematic categories for narrative synthesis: clinical care outcomes (*n* = 15), provider perspectives (*n* = 11), data migration (*n* = 8), patient experience (*n* = 4), and other topics (*n* = 5). Many studies described single sites that are early adopters of technology with robust research resources, switching from a homegrown system to a commercial system, and emphasized the dynamic effect of transitioning on important clinical care and other outcomes over time.

**Discussion:**

The published literature represents a heterogeneous mix of study designs and outcome measures, and while some of the stronger studies in this review used longitudinal approaches to compare outcomes across more sites, the current literature is primarily descriptive and is not designed to offer recommendations that can guide future EHR transitions. Transitioning from one EHR to another constitutes a major organizational change that requires nearly every person in the organization to change how they do their work. Future research should include human factors as well as diverse methodological approaches such as mixed methods and implementation science.

**Supplementary Information:**

The online version contains supplementary material available at 10.1007/s11606-023-08276-3.

## BACKGROUND

The passage of the Health Information Technology for Economic and Clinical Health (HITECH) Act in 2009 accelerated the transition from paper to electronic health record (EHR) systems across the USA, with the adoption of EHRs up to 90% for office-based physicians.^1^ HITECH also introduced the concept of “meaningful use”, which established expectations that healthcare organizations would go beyond the basic implementation of EHRs to take advantage of more advanced functionality.^2^ Not all early EHR systems, whether commercial or homegrown, were designed with the capacity to accommodate such capabilities.^3^ As a result of the needs for greater functionality, as well as factors such as institutional mergers, transitions from one EHR system to another have become increasingly common.

EHR-to-EHR transitions present different challenges compared to transitions from paper medical records to EHRs, and as such, the evidence base generated from paper-to-EHR transitions may not be directly relevant. Limited evidence exists to inform healthcare organizations, frontline providers, and other key stakeholders about EHR-to-EHR transitions. One narrative review of EHR-to-EHR transitions focused on describing challenges with the transitions, and strategies to overcome them;^4^ another review captured lessons learned from ten transitions.^5^ However, the overlap between the publications was low, and neither intended to systematically describe the literature in this area. Combined they identified 84 unique publications, but only 11 of these appeared in both publications. The majority of studies (73/84, 87%) appeared in only one or the other publication, indicating a need for comprehensive synthesis to identify and describe the published findings in this field at large.

The objective of the present review is to synthesize the entire body of peer-reviewed literature on EHR-to-EHR transitions. We used broad search criteria to include all literature from the two prior reviews, plus articles that have been published since those literature searches. We characterized the literature to describe the range of study designs, settings, and outcome measures reported to evaluate the generalizability of published work and identify knowledge gaps where more evidence is needed.

## METHODS

Our study protocol is registered with PROSPERO (CRD42021254671). We report our review according to the Preferred Reporting Items for Systematic Reviews and Meta-Analyses (PRISMA) statement (see Appendix).^6^ We used a narrative approach to synthesize findings.

### Search Strategy

Two recent reviews served as the initial literature source: a 2018 narrative review by Saleem & Herout^4^ and a 2020 review by Huang and colleagues.^5^ Saleem and Herout did not report a formal search strategy; therefore, we retrieved all publications that they included for full-text review. Huang and colleagues conducted two searches on PubMed with broad terms relating to “electronic health records” or “transition”, with search dates ending on 17 July 2020 and 10 August 2020;^5^ we again retrieved all publications included for full-text review and updated PubMed search results through 08 July 2022 (see Appendix for details).

### Study Selection

We excluded publications that did not report original data, such as commentaries, non-systematic reviews, or editorials, and studies that described transitions from paper records to an EHR.

All stages of screening and review were conducted independently in duplicate, with disagreements at abstract and full-text stages reconciled through group discussion. Eight team members reviewed the full-text publications retrieved from the existing reviews, with disagreements resolved through team discussion. Four team members screened citations identified by the updated PubMed search. Two team members screened abstracts of citations deemed relevant by at least one reviewer. The full-text review was conducted by two team members independently, with a third team member resolving conflicts as needed.

### Data Abstraction

Data extraction was completed independently in duplicate. All discrepancies were resolved with group discussion. We abstracted data on study design, setting, sample size, original EHR system, new EHR system, dates of transition, dates of data collection, outcomes reported, and key findings (see Appendix for data abstraction form). In cases where multiple publications describe the same institution’s transition but provided different dates (e.g., a go-live date in one publication and a transition date range in another), we used the earliest date provided for that transition. When available, we captured information on EHR capabilities before and after the transition (i.e., increase in capability from low capability to high capability, low capability to low capability, high capability to high capability). For example, an increase in capability occurs when a health system transitions from a locally developed, workstation-based EHR to a commercial, networked EHR.

### Data Synthesis

We grouped articles into thematic categories and narratively synthesized findings. If an article reported outcomes that spanned categories (e.g., patient experience and provider productivity), it was included in both categories.

### Funding Source

This work was supported by VA Health Services Research and Development (HSR&D) Coordinating Hub to Promote Research Optimizing Veteran-centric EHR Networks (PROVEN Hub; SDR 20–197). VA HSR&D had no role in the design, conduct, or reporting of this work.

## RESULTS

**Figure 1:**
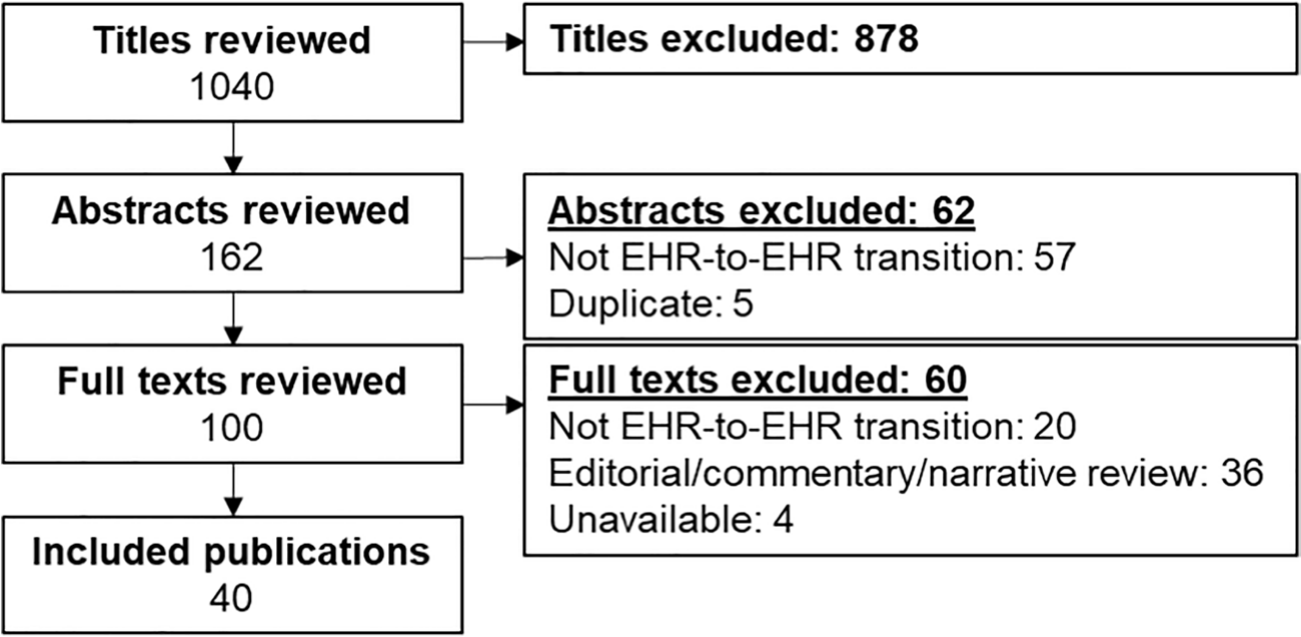
**Literature flow.**

We screened 956 unique titles generated from our search results in addition to the 84 citations from the index publications by Huang and colleagues or Saleem and Herout^4,5^ for a total of 1040 unique citations (Fig. 1). Of the 100 publications that met the criteria for full-text review after the title and abstract screening, 60 were excluded (see Appendix for excluded studies). Of the 84 citations from the two index publications, we included 26 in our final synthesis. Notably, only 6 of these appeared in both reviews, the majority (20/26, 77%) appeared in only one publication or the other. We identified 14 new publications (35% of the total included articles) that were not included in either index publication. The combined 40 included publications were sorted by topics they addressed: clinical care outcomes (*n* = 15), provider perspectives (*n* = 11), data migration (*n* = 8), patient experience (*n* = 4), and other topics (*n* = 5). Some publications addressed more than one topic.

A variety of study designs were used to examine EHR-to-EHR transitions (see Table 1) including mixed-methods (*n* = 6), quantitative-only (*n* = 30), qualitative-only (*n* = 3), and systematic review (*n* = 1). Of the six mixed-methods studies, two were longitudinal. The 30 quantitative-only analyses included time series (*n* = 12), one time point before the transition and two time points after (*n* = 5); one time point before and one after transition (*n* = 4); and post-transition only (*n* = 9). Two qualitative studies focused on post-transition; one qualitative study focused on data collected during transition. (See Evidence Table in Appendix for details of included studies.)

**Table 1:** Study Design of Included Studies (*n* = 40)

First author and year of publication	Quantitative outcome analysis				Qualitative or other analysis
	Time series	Before, and two times after, deployment	Before, and one time after, deployment	Only one time after deployment	
Mixed methods (n = 6)					
Whalen, 2018^7^	x				Analysis of errors
Dunn Lopez, 2021^8^		x			Heuristic evaluation
Pirtle, 2019^9^			x		Qualitative, 1 post-deployment time
Abramson, 2013^10^				x	Qualitative, 1 post-deployment time
Amlung, 2020^11^				x	Qualitative, 1 post-deployment time
Makar, 2014^12^				x	Qualitative, 1 post-deployment time
Quantitative outcome analysis only (n = 30)					
Barnett, 2016^13^	x				
Binney, 2020^14^	x				
Colicchio, 2018^15^	x				
Epstein, 2019^16^	x				
Friebe, 2020^17^	x				
Hanauer, 2017^18^	x				
North, 2020^19^	x				
Sivashanker, 2021^20^	x				
Tan, 2017^21^	x				
Tian, 2021^22^	x				
Wright, 2018^23^	x				
Yuan, 2021^24^	x				
Abramson, 2011^25^		x			
Calder-Sprackman, 2021^26^		x			
Krousel-Wood, 2017^27^		x			
Monturo, 2022^28^		x			
Pandit, 2013^29^		x			
McEvoy, 2018^30^			x		
Reeves, 2020^31^			x		
Zandieh, 2012^32^			x		
Zheng, 2020^33^			x		
Adler, 2015^34^				x	
Behlen, 2000^35^				x	
Lammers, 2011^36^				x	
MacKenzie, 2021^37^				x	
Michel, 2014^38^				x	
Pageler, 2016^39^				x	
Pantaleoni, 2015^40^				x	
Pfoh, 2012^41^				x	
Wang, 2020^42^				x	
Qualitative or other analysis only (n = 4)					
Abramson, 2012^43^					Qualitative, 1 post-deployment time
Abramson, 2016^44^					Qualitative, 1 post-deployment time
Umstead, 2021^45^					Qualitative, during transition
Schreiber, 2020^46^					Systematic review and case studies

### Transition Characteristics

**Figure 2:**
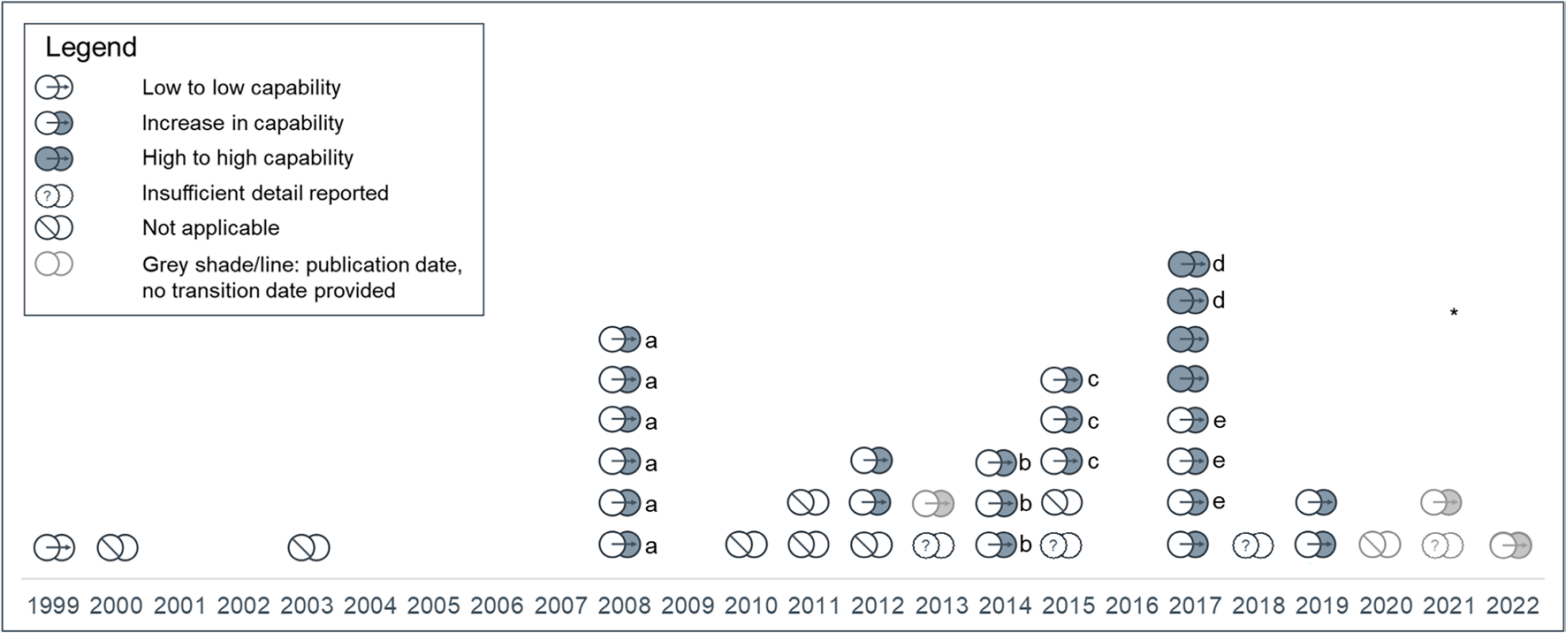
**Transition start date and capability change during transition (*n* = 40).**

Most studies reported EHR-to-EHR transitions that occurred in 2008 or later (see Fig. 2). Publications varied in how they reported transition dates (e.g., go-live date, transition period of 1 to 7 years); five publications did not report transition dates.^7–11^ Twenty-three publications described an increase in capabilities;^7,10–31^ four publications reported transitions to an EHR with capabilities similar to the legacy system.^20,32–34^ These four high-capability to high-capability transitions were in 2014 or later. The single instance of low-capability to low-capability transition was also the earliest transition identified in this review, in 1999.^35^ For the remaining 12 publications, the change in capacity was either unclear^8,36–41^ or not described.^9,42–45^

**Figure 3:**
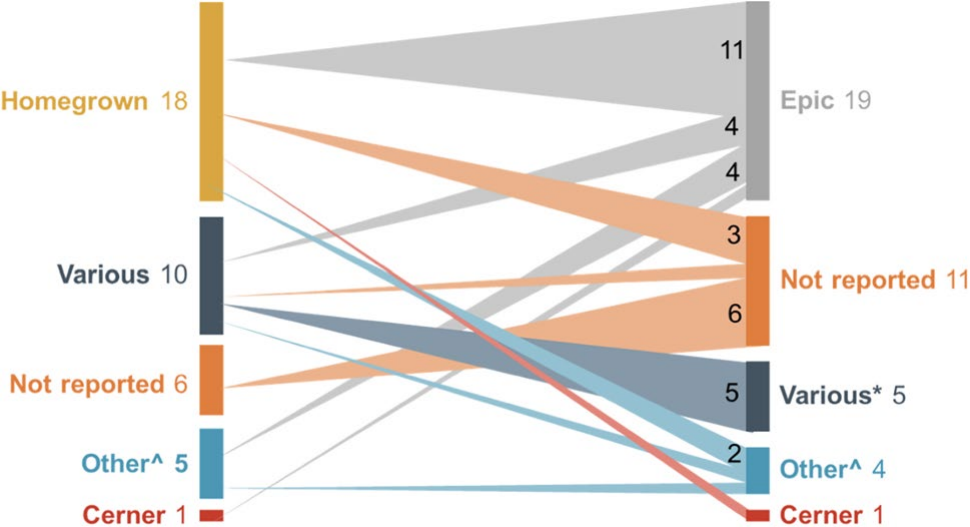
EHR systems described in included publications (*n* = 40). Number of EHR transition = 1 unless otherwise noted. *more than 1 EHR system represented. ^other EHR systems such as QuadRI and AllScripts; details in evidence table.

The plurality of publications described transitions from a homegrown EHR to Epic (see Fig. 3). Five institutions—Weill Cornell Medical College, Mass General Brigham network, Stanford Health System, Vanderbilt University Medical Center, and Mayo Clinic Health System–had multiple publications describing their EHR transitions.

### Clinical Care

Fifteen publications addressed a clinical care outcome. ^7,10,14–16,23–25,28,30,31,34,38,44,46^ These include time series designs with quantitative data (*n* = 7), pre-post design with quantitative data (*n* = 5), and mixed-methods (*n* = 3). We identified three subcategories within clinical care outcomes: quality of care, patient safety, and workflow or productivity. All but three studies were from single-site or small systems. The exceptions were: a large difference-in-difference analysis of 17 transitioning hospitals that used Medicare data;^38^ a medical-record review in 42 pediatric primary care practices;^32^ and an interrupted time series. The interrupted time series compared 41 measures of quality, patient safety, and productivity between an intervention group (four hospitals and 39 primary care clinics) that completed the transition to a new EHR and a control group (two hospitals and 10 clinics) within the same health system (Intermountain) that had not yet transitioned;^44^ data were collected monthly for 6.5 years before, during, and after the transition.

### Quality of Care

Four studies addressed quality of care outcomes,^24,32,38,44^ including the three large studies described above.^32,38,44^ The study of pediatric primary care practices focused on documentation of age-appropriate well child services. Findings showed mostly non-significant change following EHR transition in the 62 elements included in the chart review; the exception was a statistically significant improvement in the documentation related to a picklist of age-appropriate, guideline-informed practices.^32^ In the difference-in-difference Medicare study of 17 hospitals, no association was found between EHR transition and 30-day mortality rates or all-cause 30-day readmission rates, after adjusting for sociodemographic and clinical characteristics.^38^

The Intermountain study included 11 ambulatory care and hospital quality measures and evaluated patterns in each measure’s change over time, by service region, in sites undergoing the transition compared to control sites.^44^ Blood pressure control for patients with diabetes mellitus decreased significantly in the four regions where the data were available immediately after go-live and then recovered to baseline levels in two regions within seven to 16 months. Other quality of care measures showed greater variability by service region.

A fourth publication reported an interrupted time series study based on one tertiary medical center. This study reported an increase in follow-up time for one of three types of nonurgent but clinically significant test results after EHR implementation; no change in follow-up was reported for the other two tests.^24^

### Patient Safety

Seven publications included patient safety outcomes,^15,16,23,25,28,38,44^ including the Intermountain and difference-in-difference Medicare studies. The Intermountain study did not find similar patterns across sites in the 10 patient safety measures they included.^44^ The Medicare study measured adverse safety events using the PSI-90 composite measure, a set of 10 measures that were developed and refined by the Agency for Healthcare Research and Quality, are endorsed by the National Quality Forum, and are included in the Center for Medicare & Medicaid Services Bundled Payments for Care Improvement Advanced Model.^47^ The analysis did not identify any association between the EHR transition and adverse safety events.^38^

The five other publications in this category focused on medication and prescribing safety outcomes in single sites using longitudinal data.^15,16,23,25,28^ Two studies reporting on the Weill Cornell EHR transition suggest that prescribing errors were highest before go-live and decreased over the two years following implementation. Inappropriate abbreviation errors initially increased after implementation, then decreased over the remainder of the two-year study period.^15,16^ Authors noted that ongoing system refinements were a factor in this decrease. At another large academic health system, pediatric medication safety reports rose by a factor of five before returning to baseline levels three months after implementation.^25^ A study in another system reported a sixfold increase in drug-drug interaction alert burden following implementation; alert burden decreased by 51% after system changes were made to tailor the alerts.^23^ The last study in this category did not show a spike in errors; rather, prescribing rates for potentially inappropriate medications decreased after the EHR transition.^28^ These last two studies demonstrated low provider acceptance rates of alerts after implementation, ranging from 7.5 to 13%.^23,28^

### Workflow/Productivity

Seven studies measured aspects of workflow and productivity.^7,10,30,31,34,44,46^ Four of these studies evaluated time spent on clinical encounters and in the EHR using pre-post study designs with one or two post-implementation time points; all four reported negative impacts on productivity immediately after transition.^7,10,30,34^ These outcomes were consistent across the range of clinical settings included in the studies: glaucoma subspecialty practice,^7^ emergency department, ^30^ preoperative assessment,^34^ and urgent care.^10^ The study of the glaucoma subspecialty practice reported a return to baseline levels six months post-transition.^7^ Two publications reported decreased turnaround times after transition for clinical pathology tests^46^ and radiology.^31^ Some of the 20 productivity measures included in the Intermountain Healthcare study showed similar patterns of change across the transitioning regions.^44^ These included emergency department length of stay and wait times, which both increased immediately after go-live before recovering to baseline levels by one year later. New patient visits and lab test orders both decreased immediately and had not recovered by the end of the study.

### Provider Perspectives

Eleven publications reported studies about provider perspectives of EHR-to-EHR transitions.^12–19,22,27,37^ These included satisfaction and lessons learned for training providers on a new EHR (*n* = 2),^22,27^ provider satisfaction and perceptions of the new EHR (*n* = 3),^18,19,37^ and medication or prescribing safety (*n* = 6; all from Weill Cornell),^12–17^ Two of these six were mentioned in the patient safety category above.^15,16^

One study of three clinical services in a large academic healthcare system transition reported a two-year time series that looked across multiple dimensions of data entry, communication, safety, reminders and alerts, workflow and efficiency, job satisfaction, and looking forward.^18^ The authors described post-implementation changes as following various patterns such as a J-curve, L-curve, U-curve, and flat line. The most common pattern was an early decline after transition, followed by gradual improvement, but remaining below baseline at the study endpoint (i.e., L-curve pattern). Another study with two time periods after transition (6–12 and 12–24 months) similarly reported initial dips followed by recovery on most measures.^19^

A different study surveyed family physician readers of *Family Practice Management* about their satisfaction with their new EHR, key factors driving the decision to transition, and challenges that their practice faced.^37^ Respondents were split on various facets of satisfaction, with more respondents reporting that their new EHR did not improve productivity (49%) than reporting improved productivity (28%).

The six publications from Weill Cornell address various facets of provider perspectives on the transition, the new EHR, and its impact on medication safety using a few different sources of qualitative and quantitative data.^12–17^ Findings were consistent with the other studies in this category and emphasized the need for ongoing training and tailoring of the EHR system over time.

### Data Migration

Eight publications addressed data migration from the old to new EHR. Migration is achieved through exporting and then importing digital files.^9,20,26,35,42,43,45^ The eight articles reported various permutations of this process, including manual entry, automation (scripting) of “manual” data entry by computer, and mapped free-text to a standardized terminology. One publication reporting two case studies and a literature review found that no two sites used the same approach to data migration and suggested that prospective evaluation of the migration process would be helpful in this area of “very little empiric information”.^41^ Three publications discussed transitions from multiple fragmented legacy EHRs to one single system across three affiliated hospitals,^26^ a single institution,^43^ and within three Swedish counties.^42^ Three publications described specific aspects of data migration at individual hospitals, including migrating free-text allergy information,^45^ migrating radiology data,^35^ and moving from one high-capability system to another.^20^ The last study in this category reported using a participatory approach to define data migration across ambulatory clinics to understand the needs of clinical end-users.^9^

### Patient Experience

Four publications addressed patient satisfaction outcomes.^7,11,33,48^ A study conducted in a glaucoma subspecialty practice did not identify significant changes in patient satisfaction 6 months after an EHR transition.^7^ A study conducted at a community hospital found no statistically significant differences in patient experience prior to EHR transition and at 6-week and 6-month post-transition.^11^ Another publication reported a time series study conducted across outpatient services at six Mayo Clinic sites. Results showed significant reductions in patient satisfaction following an EHR transition, with a return to baseline satisfaction levels between 9 and 15 months after go-live.^33^ Patient-reported quality of care and satisfaction were less affected by the transition, whereas overall satisfaction and satisfaction with access followed a J-curve pattern, dropping initially and then exceeding baseline levels by the end of the 20-month study. Another time-series study conducted across 10 hospitals in a Midwest healthcare system reported that, after an initial decline in the first quarter following the EHR transition, patient experience scores returned to baseline by the second quarter.^48^

### Other Topics

Five publications met our inclusion criteria but did not fit into the categorizations above.^8,29,36,39,40^ Two publications reported qualitative studies that explored the transition process and lessons learned from the Indian Health Service and Vanderbilt transitions.^8,41^ Both highlighted interpersonal dynamics and the roles that trust, communication, and leadership support can play in a transition. Two other studies used quantitative analyses to ascertain key organizational characteristics that might affect EHR use. The first of these was focused on vendor switching or dropping,^36^ while the second was focused on the conversions from signing up to going live, and from going live to meaningful use.^40^ The fifth publication reported that EHR implementation did not seem to have an effect on bond ratings.^39^

## DISCUSSION

Evaluation of the impact and process of transitioning from a legacy EHR to a new EHR is an emerging area of study. Most published studies represent experiences at single sites that are early adopters of technology with robust research resources. The plurality of institutions represented in the literature switched from a homegrown EHR system to a commercial solution, thereby increasing the capabilities. This review identified specific outcomes that were positively, negatively, or not significantly associated with EHR transition in the 40 publications categorized as clinical care outcomes (*n* = 15), provider perspectives (*n* = 11), data migration (*n* = 8), patient experience (*n* = 4), and other topics (*n* = 5). Mortality and hospital readmission showed no significant association with EHR transition.^38^ Potentially inappropriate prescribing^15,16,28^ and testing turnaround time showed improvements.^31,46^ These findings illustrate some of the potential benefits of upgrading an EHR system, and may help practitioners understand the possible positive outcomes of a typically disruptive process. Outcomes that appeared at least temporarily worse—including prescribing errors,^15,16^ drug-drug interaction alerts,^23^ emergency-department waits,^44^ job satisfaction,^18,19^ and time spent in the EHR^7,10,30,34^—may require special attention as institutions undergo EHR transition.

One key theme across the literature was the importance of viewing the transition as an evolving change process, rather than the flipping of a switch. Studies in this review that used some variation of a time series study design offered insights into longitudinal changes, identifying J-, U-, or L-shaped curves for various outcomes (e.g., patient satisfaction, provider perceptions of functionality, provider satisfaction, and clinical outcomes).^18^ Such studies are important in helping health systems and providers anticipate and proactively address challenges that emerge during EHR transitions. The inclusion of qualitative methods helped identify adaptations and practices that contributed to implementation success. Taken together, both quantitative and qualitative research approaches are necessary to understand this complex, disruptive process. Employing the findings from this body of evidence can help healthcare system leaders and front-line clinicians alike know what to expect from EHR transitions, and can help implementation leaders avoid common pitfalls. This review also draws attention to the several under-explored aspects of EHR transitions that would benefit from further study.

### Future Directions

Future research on EHR transitions should take advantage of diverse methodological approaches, including human factors, mixed methods, and implementation studies. Relevant areas of focus include patient safety outcomes, data migration strategies, and workarounds, capacity for health information exchange, care coordination across specialties, clinical decision support, and individual user practices to optimize EHR transition outcomes. The Veterans Health Administration (VA) transition from a homegrown EHR to the Oracle Cerner Millennium EHR system presents a tremendous opportunity to systematically study a transition of unprecedented size and scope that could address many of these areas. Other healthcare systems planning EHR-to-EHR transitions should also consider collecting data to follow the longitudinal changes throughout the transition process.

### Limitations

Many EHR transitions occur as part of regular healthcare system operations. Even when formal evaluations are conducted, results may not be reported in the peer-reviewed literature; thus, there is potential publication bias. For instance, the Department of Defense has been undergoing a system-wide transition from a legacy EHR to MHS Genesis since 2013. Although some information is available in government reports and online, we did not identify any peer-reviewed publications about this transition.^49–51^

EHR transitions that are reported in the literature and were captured by our search strategy used a heterogeneous mix of study designs and outcome measures which made it difficult to draw strong conclusions across studies. Study designs frequently did not include longitudinal data for comparators to contextualize the changes that were described. Institutions represented in the literature may be early adopters of technology with robust research resources and therefore may not reflect the milieu of typical EHR transitions.

## CONCLUSION

Transitioning from one EHR to another constitutes a major organizational change that requires nearly every person in the organization to change their workflows. Because most healthcare systems have evolved beyond the era of paper-to-EHR transitions to expand capabilities, the evidence must now evolve to support the critical decisions facing healthcare systems and practitioners when preparing for and transitioning to a new EHR. Future studies must systematically evaluate the various aspects of EHR transitions to help institutions realize the promises of increased value to their organizations and, most importantly, improved quality of care for patients.

### Supplementary Information

Below is the link to the electronic supplementary material.

Supplementary file1 (DOCX 104 kb)

## Data Availability

All data is from publicly available literature, but data extracted and used for analysis is available from the corresponding author upon reasonable written request.

## References

[1] Centers for Disease Control and Prevention. Electronic Medical Records/Electronic Health Records (EMRs/EHRs) [Internet]. CDC; 2023 Jan [2023 Jun 27] Available from https://www.cdc.gov/nchs/fastats/electronic-medical-records.htm.

[2] Brann A, Janvanishstaporn S, Greenberg B (2020). Association of Prior Left Ventricular Ejection Fraction With Clinical Outcomes in Patients With Heart Failure With Midrange Ejection Fraction. JAMA Cardiol..

[3] Holmgren AJ, Adler-Milstein J, McCullough J (2018). Are all certified EHRs created equal? Assessing the relationship between EHR vendor and hospital meaningful use performance. J Am Med Inform Assoc..

[4] **Saleem JJ, Herout J.** Transitioning from one Electronic Health Record (EHR) to another: a narrative literature review. In Proceedings of the Human Factors and Ergonomics Society Annual Meeting 2018 Sep (Vol. 62, No. 1, pp. 489-493). Sage CA: Los Angeles, CA: SAGE Publications.

[5] Huang C, Koppel R, McGreevey JD (2020). Transitions from One Electronic Health Record to Another: Challenges, Pitfalls, and Recommendations. Appl Clin Inform..

[6] Page MJ, McKenzie JE, Bossuyt PM (2021). The PRISMA 2020 statement: an updated guideline for reporting systematic reviews. BMJ..

[7] Pandit RR, Boland MV (2013). The impact of an electronic health record transition on a glaucoma subspecialty practice. Ophthalmology..

[8] Amlung J, Huth H, Cullen T, Sequist T (2020). Modernizing health information technology: lessons from healthcare delivery systems. JAMIA Open..

[9] MacKenzie B, Anaya G, Hu J (2021). Defining Data Migration Across Multidisciplinary Ambulatory Clinics Using Participatory Design. Appl Clin Inform..

[10] Dunn Lopez K, Chin CL, LeitãoAzevedo RF (2021). Electronic health record usability and workload changes over time for provider and nursing staff following transition to new EHR. Appl Ergon..

[11] Monturo C, Brockway C, Ginev A (2022). Electronic Health Record Transition: The Patient Experience. Comput Inform Nurs: CIN..

[12] Zandieh SO, Abramson EL, Pfoh ER (2012). Transitioning between ambulatory EHRs: a study of practitioners' perspectives. J Am Med Inform Assoc..

[13] Pfoh ER, Abramson E, Zandieh S (2012). Satisfaction after the transition between electronic health record systems at six ambulatory practices. J Eval Clin Pract..

[14] Abramson EL, Patel V, Malhotra S (2012). Physician experiences transitioning between an older versus newer electronic health record for electronic prescribing. Int J Med Inform..

[15] Abramson EL, Malhotra S, Fischer K (2011). Transitioning between electronic health records: effects on ambulatory prescribing safety. J Gen Intern Med..

[16] Abramson EL, Malhotra S, Osorio SN (2013). A long-term follow-up evaluation of electronic health record prescribing safety. J Am Med Inform Assoc..

[17] Abramson EL, Patel V, Pfoh ER, Kaushal R (2016). How Physician Perspectives on E-Prescribing Evolve over Time. A Case Study Following the Transition between EHRs in an Outpatient Clinic. Appl Clin Inform..

[18] Hanauer DA, Branford GL, Greenberg G (2017). Two-year longitudinal assessment of physicians' perceptions after replacement of a longstanding homegrown electronic health record: does a J-curve of satisfaction really exist?. J Am Med Inform Assoc..

[19] **Krousel-Wood M, McCoy AB, Ahia C, et al.** Implementing electronic health records (EHRs): health care provider perceptions before and after transition from a local basic EHR to a commercial comprehensive EHR. J Am Med Inform Assoc 2018;25(6):618-26. 10.1093/jamia/ocx094 PMC7646958 29036503

[20] Pageler NM, GrazierG’Sell MJ, Chandler W (2016). A rational approach to legacy data validation when transitioning between electronic health record systems. J Am Med Inform Assoc.

[21] Koppel R, Lehmann CU (2014). Implications of an emerging EHR monoculture for hospitals and healthcare systems. J Am Med Inform Assoc..

[22] Pantaleoni JL, Stevens LA, Mailes ES (2015). Successful physician training program for large scale EMR implementation. Appl Clin Inform..

[23] Wright A, Aaron S, Seger DL (2018). Reduced Effectiveness of Interruptive Drug-Drug Interaction Alerts after Conversion to a Commercial Electronic Health Record. J Gen Intern Med..

[24] Sivashanker K, Bell G, Khorasani R (2021). Electronic Health Record Transition and Impact on Screening Test Follow-Up. Jt Comm J Qual Patient Saf..

[25] Whalen K, Lynch E, Moawad I (2018). Transition to a new electronic health record and pediatric medication safety: lessons learned in pediatrics within a large academic health system. J Am Med Inform Assoc..

[26] Epstein RH, Dexter F, Schwenk ES (2019). Provider Access to Legacy Electronic Anesthesia Records Following Implementation of an Electronic Health Record System. J Med Syst..

[27] Pirtle CJ, Reeder RR, Lehmann CU (2019). Physician Perspectives on Training for an EHR Implementation. Stud Health Technol Inform..

[28] Friebe MP, LeGrand JR, Shepherd BE (2020). Reducing Inappropriate Outpatient Medication Prescribing in Older Adults across Electronic Health Record Systems. Appl Clin Inform..

[29] Umstead CN, Unertl KM, Lorenzi NM, Novak LL (2021). Enabling adoption and use of new health information technology during implementation: Roles and strategies for internal and external support personnel. J Am Med Inform Assoc..

[30] Calder-Sprackman S, Clapham G, Kandiah T (2021). The impact of adoption of an electronic health record on emergency physician work: A time motion study. J Am Coll Emerg Physicians Open..

[31] **Reeves JJ, Longhurst CA, San Miguel SJ, et al.** Bringing student health and Well-Being onto a health system EHR: the benefits of integration in the COVID-19 era. J Am Coll Health. 2020:1–7. doi:10.1080/07448481.2020.1843468. 10.1080/07448481.2020.1843468 33180683

[32] Binney G, Cole-Poklewski T, Roomian T (2020). Effect of an electronic health record transition on the provision of recommended well child services in pediatric primary care practices. Clin Pediatr (Phila).

[33] North F, Pecina JL, Tulledge-Scheitel SM (2020). Is a switch to a different electronic health record associated with a change in patient satisfaction?. J Am Med Inform Assoc..

[34] Zheng L, Duncan BJ, Kaufman DR (2020). EHR Conversion on the PreOp Care: A Pre-Post Workflow Comparison. AMIA Annu Symp Proc..

[35] Behlen FM, Sayre RE, Weldy JB, Michael JS (2000). "Permanent" records: experience with data migration in radiology information system and picture archiving and communication system replacement. J Digit Imaging..

[36] Lammers EJ, Zheng K (2011). Characteristics associated with hospital health IT vendor switching and dropping. AMIA Annu Symp Proc..

[37] Adler KG, Edsall RL (2015). EHR Switch Survey: Responses From 305 Family Physicians. Fam Pract Manag..

[38] **Barnett ML, Mehrotra A, Jena AB.** Adverse inpatient outcomes during the transition to a new electronic health record system: observational study. BMJ. 2016 Jul 28;354. 10.1136/bmj.i3835 PMC4964115 27471242

[39] McEvoy D, Barnett ML, Sittig DF (2018). Changes in hospital bond ratings after the transition to a new electronic health record. J Am Med Inform Assoc..

[40] Yuan B, Li J, Wu P (2021). The effectiveness of electronic health record promotion for healthcare providers in the United States since the Health Information Technology for Economic and Clinical Health Act: An empirical investigation. Int J Health Plann Manage..

[41] Schreiber R, Garber L (2020). Data migration: a thorny issue in electronic health record transitions—case studies and review of the literature. ACI Open..

[42] **Makar M.** Dealing with existing data in legacy systems when transitioning between Electronic Health Records in three Swedish counties. Accessed 21 Aug 2020, https://ki.se/sites/default/files/migrate/dealing_mina_makar.pdf.

[43] Michel J, Hsiao A, Fenick A (2014). Using a scripted data entry process to transfer legacy immunization data while transitioning between electronic medical record systems. Appl Clin Inform..

[44] Colicchio TK, Del Fiol G, Scammon DL (2018). Comprehensive methodology to monitor longitudinal change patterns during EHR implementations: a case study at a large health care delivery network. J Biomed Inform..

[45] Wang AY, Osborne JD, Danila MI (2020). AllergyMap: An Open Source Corpus of Allergy Mention Normalizations. AMIA Annu Symp Proc..

[46] Tan BT, Fralick J, Flores W (2017). Implementation of Epic Beaker Clinical Pathology at Stanford University Medical Center. Am J Clin Pathol..

[47] Farquhar M. AHRQ Quality Indicators. In: Hughes RG, editors. Vol. 3 Patient Safety and Quality: An Evidence-Based Handbook for Nurses. Rockville: Agency for Healthcare Research and Quality; 2008. p. 41-67. 21328752

[48] Tian D, Hoehner CM, Woeltje KF (2021). Disrupted and Restored Patient Experience With Transition to New Electronic Health Record System. J Patient Experience..

[49] Department of Veterans Affairs. Veterans Health Administration: About the VHA [Internet]. US Department of Veterans Affairs; 2023 Jun [2023 Jun 27]. Available from: https://www.va.gov/health/aboutvha.asp.

[50] Military Health System. Genesis of MHS GENESIS [Internet]. Health.mil; 2023 Jun [2023 Jun 27]. Available from: https://www.health.mil/Military-Health-Topics/Technology/MHS-GENESIS/MHS-GENESIS-Timeline.

[51] Mendez BH. MHS Genesis: Background and Issues for Congress. LIBRARY OF CONGRESS WASHINGTON, DC. 2019.

