# Supplementary material for: Transitioning from One Electronic Health Record to Another: A Systematic Review
Source: J Gen Intern Med. 2023 Oct 5;38(Suppl 4):956–64. doi: 10.1007/s11606-023-08276-3 (PMC10593710; doi:10.1007/s11606-023-08276-3)
Supplement: Supplementary file 1 — Supplementary file1 (DOCX 104 kb) [file 11606_2023_8276_MOESM1_ESM.docx]

**Appendix A. PRSIMA Checklist**

| **Section and Topic** | **Item #** | **Checklist item** | **Location where item is reported** |
| --- | --- | --- | --- |
| **TITLE** | | |  |
| Title | 1 | Identify the report as a systematic review. | 1 |
| **ABSTRACT** | | |  |
| Abstract | 2 | See the PRISMA 2020 for Abstracts checklist. | 3 |
| **INTRODUCTION** | | |  |
| Rationale | 3 | Describe the rationale for the review in the context of existing knowledge. | 5-6 |
| Objectives | 4 | Provide an explicit statement of the objective(s) or question(s) the review addresses. | 5 |
| **METHODS** | | |  |
| Eligibility criteria | 5 | Specify the inclusion and exclusion criteria for the review and how studies were grouped for the syntheses. | 6-7 |
| Information sources | 6 | Specify all databases, registers, websites, organisations, reference lists and other sources searched or consulted to identify studies. Specify the date when each source was last searched or consulted. | 6 |
| Search strategy | 7 | Present the full search strategies for all databases, registers and websites, including any filters and limits used. | Appendix |
| Selection process | 8 | Specify the methods used to decide whether a study met the inclusion criteria of the review, including how many reviewers screened each record and each report retrieved, whether they worked independently, and if applicable, details of automation tools used in the process. | 7 |
| Data collection process | 9 | Specify the methods used to collect data from reports, including how many reviewers collected data from each report, whether they worked independently, any processes for obtaining or confirming data from study investigators, and if applicable, details of automation tools used in the process. | 7 |
| Data items | 10a | List and define all outcomes for which data were sought. Specify whether all results that were compatible with each outcome domain in each study were sought (e.g. for all measures, time points, analyses), and if not, the methods used to decide which results to collect. | 7 |
|  | 10b | List and define all other variables for which data were sought (e.g. participant and intervention characteristics, funding sources). Describe any assumptions made about any missing or unclear information. | 7 |
| Study risk of bias assessment | 11 | Specify the methods used to assess risk of bias in the included studies, including details of the tool(s) used, how many reviewers assessed each study and whether they worked independently, and if applicable, details of automation tools used in the process. | n/a |
| Effect measures | 12 | Specify for each outcome the effect measure(s) (e.g. risk ratio, mean difference) used in the synthesis or presentation of results. | n/a |
| Synthesis methods | 13a | Describe the processes used to decide which studies were eligible for each synthesis (e.g. tabulating the study intervention characteristics and comparing against the planned groups for each synthesis (item #5)). | 7 |
|  | 13b | Describe any methods required to prepare the data for presentation or synthesis, such as handling of missing summary statistics, or data conversions. | 7 |
|  | 13c | Describe any methods used to tabulate or visually display results of individual studies and syntheses. | n/a |
|  | 13d | Describe any methods used to synthesize results and provide a rationale for the choice(s). If meta-analysis was performed, describe the model(s), method(s) to identify the presence and extent of statistical heterogeneity, and software package(s) used. | 8 |
|  | 13e | Describe any methods used to explore possible causes of heterogeneity among study results (e.g. subgroup analysis, meta-regression). | n/a |
|  | 13f | Describe any sensitivity analyses conducted to assess robustness of the synthesized results. | n/a |
| Reporting bias assessment | 14 | Describe any methods used to assess risk of bias due to missing results in a synthesis (arising from reporting biases). | n/a |
| Certainty assessment | 15 | Describe any methods used to assess certainty (or confidence) in the body of evidence for an outcome. | n/a |
| **RESULTS** | | |  |
| Study selection | 16a | Describe the results of the search and selection process, from the number of records identified in the search to the number of studies included in the review, ideally using a flow diagram. | 8 |
|  | 16b | Cite studies that might appear to meet the inclusion criteria, but which were excluded, and explain why they were excluded. | 9 (figure) |
| Study characteristics | 17 | Cite each included study and present its characteristics. | 11 |
| Risk of bias in studies | 18 | Present assessments of risk of bias for each included study. | n/a |
| Results of individual studies | 19 | For all outcomes, present, for each study: (a) summary statistics for each group (where appropriate) and (b) an effect estimate and its precision (e.g. confidence/credible interval), ideally using structured tables or plots. | 11 |
| Results of syntheses | 20a | For each synthesis, briefly summarise the characteristics and risk of bias among contributing studies. | 12-20 |
|  | 20b | Present results of all statistical syntheses conducted. If meta-analysis was done, present for each the summary estimate and its precision (e.g. confidence/credible interval) and measures of statistical heterogeneity. If comparing groups, describe the direction of the effect. | n/a |
|  | 20c | Present results of all investigations of possible causes of heterogeneity among study results. | n/a |
|  | 20d | Present results of all sensitivity analyses conducted to assess the robustness of the synthesized results. | n/a |
| Reporting biases | 21 | Present assessments of risk of bias due to missing results (arising from reporting biases) for each synthesis assessed. | n/a |
| Certainty of evidence | 22 | Present assessments of certainty (or confidence) in the body of evidence for each outcome assessed. | n/a |
| **DISCUSSION** | | |  |
| Discussion | 23a | Provide a general interpretation of the results in the context of other evidence. | 20-21 |
|  | 23b | Discuss any limitations of the evidence included in the review. | 22-23 |
|  | 23c | Discuss any limitations of the review processes used. | 22 |
|  | 23d | Discuss implications of the results for practice, policy, and future research. | 21-22 |
| **OTHER INFORMATION** | | |  |
| Registration and protocol | 24a | Provide registration information for the review, including register name and registration number, or state that the review was not registered. | 4 |
|  | 24b | Indicate where the review protocol can be accessed, or state that a protocol was not prepared. | 4 |
|  | 24c | Describe and explain any amendments to information provided at registration or in the protocol. | n/a |
| Support | 25 | Describe sources of financial or non-financial support for the review, and the role of the funders or sponsors in the review. | 1, 4 |
| Competing interests | 26 | Declare any competing interests of review authors. | 4 |
| Availability of data, code and other materials | 27 | Report which of the following are publicly available and where they can be found: template data collection forms; data extracted from included studies; data used for all analyses; analytic code; any other materials used in the review. | 4 |

*From:*  Page MJ, McKenzie JE, Bossuyt PM, Boutron I, Hoffmann TC, Mulrow CD, et al. The PRISMA 2020 statement: an updated guideline for reporting systematic reviews. BMJ 2021;372:n71. doi: 10.1136/bmj.n71

**Appendix B. Search Strategy**

Query 1

Database: PubMed

Dates: 8/11/2020 to 5/12/2021

(((“Medical Order Entry Systems”[MeSH]) OR ((((((((((“Electronic Health Records”[MeSH]) OR EHR_)

OR “electronic health record”) OR “electronic health records”) OR “electronic medical record”) OR

“electronic medical records”) OR EMR_) OR CPOE) OR “computerized physician order entry”) OR

“computerized provider order entry”))) AND ((migration_) OR transition_)

Query 2
Database: PubMed

Dates: 7/18/2020 to 5/12/2021

(((conversion) OR (convert_)) OR (switch_)) AND ((((((((((((EMR) OR (EHR)) OR (medical order entry

systems[MeSH Terms])) OR (“computerized physician order entry”)) OR (“computerized provider

order entry”)) OR (computerized physician order entry system[MeSH terms])) OR (CPOE)) OR

(“electronic health record”)) OR (“electronic health records”)) OR (“electronic medical records”))

OR (“electronic medical record”)) OR (electronic health record[MeSH Terms]))

**Appendix C. Full text data abstraction form**

1. Study design

Quantitative:

- Time series
- Before, and two times after, deployment
- Before, and one time after, deployment
- One time after deployment
  - Qualitative: _________________
  - Other: ______________________

2. Setting (name & # of sites): ________________

3. Original EHR to New EHR

Original EHR: ________________

New EHR: ________________

4. When did transition occur (i.e. go-live date): ________________

5. When did data collection occur (date or date range): ________________

6. Capacity

- low to low
- low to high
- high to hit
- unreported

7. Sample size: ________________

8. Country: ________________

**Appendix D. Excluded studies (n=60)**

**Editorial/Commentary (n=40)**

1. What is the Average IT Consulting Rate in DC, MD, and VA? Accessed August 21, 2020, https://resource.optimalnetworks.com/blog/2015/01/15/it-consulting-rate-cost-dc-md-va

2. Medical Record Shredding Guidelines for New York. Accessed 2020 August 21, https://www.confidata.com/news/paper-shredding/medical-record-shredding-guidelines/

3. Architecture. HFRURwSaS-o. Accessed August 21, 2020, http://hl7.org/fhir/services.html

4. Barkholz D. Vanderbilt is a case study for the dreaded EHR conversion. *Mod Healthc*. May 2017;47(19):10-11.

5. Bentley T, Rizer M, McAlearney AS, et al. The journey from precontemplation to action: Transitioning between electronic medical record systems. *Health Care Manage Rev*. Jan-Mar 2016;41(1):22-31. doi:10.1097/HMR.0000000000000041

6. Bornstein S. An integrated EHR at Northern California Kaiser Permanente: pitfalls, challenges, and benefits experienced in transitioning. *Appl Clin Inform*. 2012;3(3):318-25. doi:10.4338/ACI-2012-03-RA-0006

7. Center H. Planning for EHR Transition: What are your motivations for transitioning to a new electronic health record? [Technical Report]. The HITEQ Center, for Health Information Technology, Evaluation, and Quality Improvement.

8. Center H. Planning for EHR Transition: What are your motivations for transitioning to a new electronic health record? [Infographic]. . The HITEQ Center, for Health Information Technology, Evaluation, and Quality Improvement.

9. Cohen J. I'd like to phone a friend: CIOs favor peer input over rankings when choosing an EHR system. Accessed August 21, 2020, https://www.pressreader.com/usa/modern-healthcare/20190422/281900184609872

10. Collaborative KTA. Accessed August 21, 2020, http://klasresearch.com/arch-collaborative

11. Duke JD, Morea J, Mamlin B, et al. Regenstrief Institute's Medical Gopher: a next-generation homegrown electronic medical record system. *Int J Med Inform*. Mar 2014;83(3):170-9. doi:10.1016/j.ijmedinf.2013.11.004

12. Evans RS. Electronic Health Records: Then, Now, and in the Future. *Yearb Med Inform*. May 20 2016;Suppl 1:S48-61. doi:10.15265/IYS-2016-s006

13. Gettinger A, Csatari A. Transitioning from a legacy EHR to a commercial, vendor-supplied, EHR: one academic health system's experience. *Appl Clin Inform*. 2012;3(4):367-76. doi:10.4338/ACI-2012-04-R-0014

14. IT. H. Top 10 Tips for Cybersecurity in Health Care. Accessed August 21, 2020, https://www.healthit.gov/sites/default/files/Top_10_Tips_for_Cybersecurity.pdf

15. Jayanthi A EA. 8 hospitals' finances hurt by EHR costs. Becker's Hospital CFO Report. Accessed August 21, 2020, https://www.beckershospitalreview.com/finance/8-hospitals-finances-hurt-by-ehr-costs.html

16. Johnson KB, Sternberg P, Jr., Dubree M. An EPIC Switch: Observations and Opportunities After Go-Live. *J Med Syst*. Aug 11 2018;42(9):174. doi:10.1007/s10916-018-1023-5

17. Jones H. *Exploring HIT Contract Cadavers to Avoid HIT Managerial Malpractice - In: HIT or Miss: Lessons Learned from Health Information Technology Implementations*. AHIMA Press; 2019.

18. Jones S KR, Ridgely M. . Guide to Reducing Unintended Consequences of Electronic Health Records. . Accessed September 21, 2020, https://digital.ahrq.gov/sites/default/files/docs/publication/guide-to-reducing-unintended-consequences-of-electronic-health-records.pdf

19. Kannry J, Sengstack P, Thyvalikakath TP, et al. The Chief Clinical Informatics Officer (CCIO): AMIA Task Force Report on CCIO Knowledge, Education, and Skillset Requirements. *Appl Clin Inform*. 2016;7(1):143-76. doi:10.4338/ACI-2015-12-R-0174

20. Koppel R. Great promises of healthcare information technology deliver less. *Healthcare Information Management Systems: Cases, Strategies, and Solutions*. Springer International Publishing; 2016:101-125.

21. Koppel R TH. Lessons fromthe 100 Nation Ransomware Attack. Accessed August 21, 2020, https://thehealthcareblog.com/blog/2017/05/14/lessons-from-the-100-nation-ransomware-attack/.

22. Lin J, Ranslam K, Shi F, Figurski M, Liu Z. Data Migration from Operating EMRs to OpenEMR with Mirth Connect. *Stud Health Technol Inform*. 2019;257:288-292.

23. M. R. Will 2018 be the year healthcare addresses its turnover problem? . Becker’s Hospital CFO Report. https://www.beckershospitalreview.com/finance/will-2018-bethe-year-healthcare-addresses-its-turnover-problem.html

24. Mandel JC, Kreda DA, Mandl KD, Kohane IS, Ramoni RB. SMART on FHIR: a standards-based, interoperable apps platform for electronic health records. *J Am Med Inform Assoc*. Sep 2016;23(5):899-908. doi:10.1093/jamia/ocv189

25. Murphy KAA, 2020. Epic EHR Adoption Partly to Blame in Maine Hospital Debate. EHR Intelligence.. . Accessed August 21, 2020, https://ehrintelligence.com/news/epic-ehr-adoption-partly-to-blame-in-maine-hospital-debate

26. NJ G. DRESSed for failure. *AORN J*. 2015;101(4)

27. Penrod LE. Electronic Health Record Transition Considerations. *PM R*. May 2017;9(5S):S13-S18. doi:10.1016/j.pmrj.2017.01.009

28. Price S. Making the CEHRT Switch: EHR Upgrade Required for Incentive Payment Programs. *Tex Med*. Jan 1 2019;115(1):40-42.

29. R. C. Medical Record Retention. The Doctors Company. Accessed August 21, 2020, https://www.thedoctors.com/articles/medical-recordretention/

30. Ray JM, Ratwani RM, Sinsky CA, et al. Six habits of highly successful health information technology: powerful strategies for design and implementation. *J Am Med Inform Assoc*. Oct 1 2019;26(10):1109-1114. doi:10.1093/jamia/ocz098

31. Saleem JJ, Herout J. Transitioning from one Electronic Health Record (EHR) to another: a narrative literature review. SAGE Publications Sage CA: Los Angeles, CA; 2018:489-493.

32. Sittig DF, Wright A, Ash J, Singh H. New Unintended Adverse Consequences of Electronic Health Records. *Yearb Med Inform*. Nov 10 2016;(1):7-12. doi:10.15265/IY-2016-023

33. Walker J KR. For healthcare cybersecurity the whole is weaker than the sum of the parts. Accessed August 21, 2020, https://thehealthcareblog.com/blog/2016/09/23/for-healthcare-cybersecurity-thewhole-is-weaker-than-the-sum-of-the-parts/.

34. Wilson L. EHR DO-OVER? Providers begin replacing electronic patient record systems. *Health Data Manag*. Feb 2017;25(1):56-58.

35. Wisniewski PJ KB, Lipford HR. Making privacy personal: Profiling social network users to inform privacy education and nudging. *Int J Hum Comput Stud*. 2017;98:95–108.

36. Woody Ii EW. MHS Genesis Implementation: Strategies in Support of Successful EHR Conversion. *Mil Med*. Sep 18 2020;185(9-10):e1520-e1527. doi:10.1093/milmed/usaa184

**Not EHR-to-EHR transition (n=16)**

37. Adler-Milstein J, Everson J, Lee SY. EHR Adoption and Hospital Performance: Time-Related Effects. *Health Serv Res*. Dec 2015;50(6):1751-71. doi:10.1111/1475-6773.12406

38. Bortis G. Experiences with Mirth: an open source health care integration engine. *Proceedings of the 30th International Conference on Software Engineering ICSE ’08 Association for Computing Machinery*. 2008:649–652.

39. Braverman JA, Blumenthal-Barby JS. Assessment of the sunk-cost effect in clinical decision-making. *Soc Sci Med*. Jul 2012;75(1):186-92. doi:10.1016/j.socscimed.2012.03.006

40. Camacho Rodriguez JC, Staubert S, Lobe M. Automated Import of Clinical Data from HL7 Messages into OpenClinica and tranSMART Using Mirth Connect. *Stud Health Technol Inform*. 2016;228:317-21.

41. Classen DC, Resar R, Griffin F, et al. 'Global trigger tool' shows that adverse events in hospitals may be ten times greater than previously measured. *Health Aff (Millwood)*. Apr 2011;30(4):581-9. doi:10.1377/hlthaff.2011.0190

42. Craven CK, Sievert MC, Hicks LL, Alexander GL, Hearne LB, Holmes JH. CAH to CAH: EHR implementation advice to critical access hospitals from peer experts and other key informants. *Appl Clin Inform*. 2014;5(1):92-117. doi:10.4338/ACI-2013-08-RA-0066

43. D'Amore JD, Mandel JC, Kreda DA, et al. Are Meaningful Use Stage 2 certified EHRs ready for interoperability? Findings from the SMART C-CDA Collaborative. *J Am Med Inform Assoc*. Nov-Dec 2014;21(6):1060-8. doi:10.1136/amiajnl-2014-002883

44. G. K. *Committing leadership resources: A CMIO and CPOE governance. In: Leviss J, Charney P, Corbit C, eds.* . HIT or Miss: Lessons Learned fromHealth Information Technology Implementations. AHIMA Press; 2019.

45. Koppel RL, C. U. Implications of an emerging EHR monoculture for hospitals and healthcare systems. *Journal of the American Medical Informatics Association*. 2014;22(2):465-471.

46. Magrabi F, Liaw ST, Arachi D, Runciman W, Coiera E, Kidd MR. Identifying patient safety problems associated with information technology in general practice: an analysis of incident reports. *BMJ Qual Saf*. Nov 2016;25(11):870-880. doi:10.1136/bmjqs-2015-004323

47. McGreevey JD, 3rd, Mallozzi CP, Perkins RM, Shelov E, Schreiber R. Reducing Alert Burden in Electronic Health Records: State of the Art Recommendations from Four Health Systems. *Appl Clin Inform*. Jan 2020;11(1):1-12. doi:10.1055/s-0039-3402715

48. Noblin A, Cortelyou-Ward K, Cantiello J, et al. EHR implementation in a new clinic: a case study of clinician perceptions. *J Med Syst*. Aug 2013;37(4):9955. doi:10.1007/s10916-013-9955-2

49. Patterson ESA, S.; Moffatt-Bruce, S. . Clustering and Prioritizing Patient Safety Issues during EHR Implementation and Upgrades in Hospital Settings. *Proceedings of the International Symposium on Human Factors and Ergonomics in Health Care*. 2017;6(1):125-131.

50. Phelps A, Cournoyer K, Kelting T, DeNino W, Sauer W, Jara C. Development of an Electronic Medical Record for Extracorporeal Membrane Oxygenation and Its Role in a Multidisciplinary Team. *J Extra Corpor Technol*. Sep 2020;52(3):227-236. doi:10.1182/ject-2000022

51. Reynolds TL CB, Rudkin SE, et al. . Migrating from one comprehensive commercial EHR to another: perceptions of front-line clinicians and staff. . *AMIA Annu Symp Proc*. 2020:765–773.

52. Schwarz M, Coccetti A, Draheim M, Gordon G. Perceptions of allied health staff of the implementation of an integrated electronic medical record across regional and metropolitan settings. *Aust Health Rev*. Dec 2020;44(6):965-972. doi:10.1071/ah19024

53. Sieja A, Markley K, Pell J, et al. Optimization Sprints: Improving Clinician Satisfaction and Teamwork by Rapidly Reducing Electronic Health Record Burden. *Mayo Clin Proc*. May 2019;94(5):793-802. doi:10.1016/j.mayocp.2018.08.036

54. Silverman HD, Steen EB, Carpenito JN, Ondrula CJ, Williamson JJ, Fridsma DB. Domains, tasks, and knowledge for clinical informatics subspecialty practice: results of a practice analysis. *J Am Med Inform Assoc*. Jul 1 2019;26(7):586-593. doi:10.1093/jamia/ocz051

55. Yuan N, Dudley RA, Boscardin WJ, Lin GA. Electronic health records systems and hospital clinical performance: a study of nationwide hospital data. *J Am Med Inform Assoc*. Oct 1 2019;26(10):999-1009. doi:10.1093/jamia/ocz092

56. Zandieh SO, Yoon-Flannery K, Kuperman GJ, Langsam DJ, Hyman D, Kaushal R. Challenges to EHR implementation in electronic- versus paper-based office practices. *J Gen Intern Med*. Jun 2008;23(6):755-61. doi:10.1007/s11606-008-0573-5

**Unavailable (n=4)**

57. Schreiber R KR, Craven C, McGreevey J. What could go wrong? Migrating from one EHR to another. In: Proc PatAAS, editor. San Francisco, CA2015.

58. Yang Y ZY. Application of Mirth Connect interface integration engine in medical data transmission. . *Industrial Control Computer* 2016;29:112–113.

59. Zandieh SO, Mills SA, Yoon-Flannery K, Kuperman GJ, Kaushal R. Providers' expectations of ambulatory electronic health records (EHRs). *AMIA Annu Symp Proc*. Nov 6 2008:1191.

60. Zandieh SO Y-FK, Yoon-Flannery K, Kuperman GJ, Hyman D, Kaushal R. . Correlates of expected satisfaction with electronic health records in office practices by practitioners. *AMIA Annu Symp Proc*. November 6, 2008;

| **Appendix E. Evidence table of included studies (n=40)** | | |
| --- | --- | --- |
| \| **CLINICAL CARE: *Quality of Care* (n=4)** \| \| \| \| --- \| --- \| --- \| \| **Author, year**  **Previous EHR**  **New HER**  **Timing of transition** \| **Setting (Country)**  **Sample size (response rate)**  **Study design: Timing of data collection** \| **Findings from abstract** \| \| Barnett, 2016^1^  Various  Not reported  2011 to 2012 \| hospitals that implemented a new inpatient EHR in 2011-21 with a single verifiable "go live" date" (USA)  Hospitals (n=416)  Time series:  2011 to 2012 \| “Before and after implementation, characteristics of admissions were similar in both study and control hospitals. Among study hospitals, unadjusted 30 day mortality (6.74% to 7.15%, P=0.06) and adverse safety event rates (10.5 to 11.4 events per 1000 admissions, P=0.34) did not significantly change after implementation of EHRs. There was an unadjusted decrease in 30 day readmission rates, from 19.9% to 19.0% post-implementation (P=0.02). In difference-indifferences analysis, however, there was no significant change in any outcome between pre-implementation and post-implementation periods (all P≥0.13).” \| \| Binney, 2020^2^  eClinicalWorks  Epic  March 2017 and June 2018 \| Pediatric Physicians’ Organization at Children’s (affiliated with Boston Children's Hospital), an independent practice association of 84 privately owned pediatric practices with over 400 primary care clinicians (USA)  Patient Charts (n=675) from 27 primary care practices  Time series:  12 Weeks before implementation, 6 weeks implementation, and 12 weeks post implementation \| “In the pretransition period, 84.5% of all recommended elements were documented versus 86.4% posttransition (P = .04). Documentation of age-appropriate anticipatory guidance showed significant positive change (69.0% to 80.2%, P = .005), but it was the only subdomain with a statistically significant increase. These increases suggest that EHR transitions have the opportunity to affect the delivery of preventive care.” \| \| Colicchio, 2018^3^  Homegrown  Cerner  February 2015 to October 2016 \| Intermountain Healthcare, a no-for-profit, integrated care delivery system of 22 hospitals and over 185 clinics covering Utah and southern Idaho (USA)  Hospitals (n=6) and Primary care clinics (n=49) in 5 regions  Time series:  February 2013 to July 2017 \| “The proposed methodology was successfully implemented and significant changes were observed in most measured variables. A significant change attributable to the intervention was observed in 12 (29%) measures in three or more regions; in 32 (78%) measures in two or more regions; and in 40 (98%) measures in at least one region. A similar pattern (i.e., same impact in three or more regions) was detected for nine (22%) measures, a mixed pattern (i.e., same impact in two regions, and different impact in other regions) was detected for nine (22%) measures, and an inconsistent pattern (i.e., did not detect the same impact across regions) was detected for 23 (56%) measures.” \| \| Sivashanker, 2021^4^  Homegrown  Epic  May 30, 2015 \| Primary care clinics affiliated with Brigham and Women's Hosptial, a tertiary care hospital (USA)  Primary care patients with following labs--Incidental lung nodules: pre (n=285), intervention (n=94), post (n=768); Pap smears: pre (n=1,510), intervention (n=278), post (n=1,601); PSA: pre (n=994), intervention (n=174), post (n=494)  Time series:  January 2013 to June 2018 \| “There were no significant differences in follow-up in the IPN or the Pap smear ITS models. In the PSA ITS model, follow-up was significantly decreasing ( p = 0.0133) in the preintervention period, and there was a significant change in trend from intervention to postintervention ( p = 0.0279).” \| \| **CLINICAL CARE: *Patient Safety* (n=7)** \| \| \| \| **Author, year**  **Previous EHR**  **New HER**  **Timing of transition** \| **Setting (Country)**  **Sample size (response rate)**  **Study design: Timing of data collection** \| **Findings from abstract** \| \| Abramson, 2011^5^  Homegrown  Epic  April 2008 \| Weill Medical College of Cornell University, academically-affiliated, hosptial-based adult internal medicine ambulatory practice in New York City (USA)  Physicians (n=17, 79%); prescriptions review: Baseline (n=646), 12-weeks (n=735), 1 year (n=715)  Quantitative, before and two times after deployment:  Survey: January to June 2009; Prescription collection: April 2008 to April 2009 \| “We analyzed 1298 prescriptions at baseline, 1331 prescriptions 12 weeks post-implementation, and 1303 prescriptions one year post-implementation. Overall prescribing error rates were highest at baseline (35.7 per 100 prescriptions, 95% confidence interval (CI) 23.2–54.8) and lowest one year postimplementation (12.2 per 100 prescriptions, 95% CI 8.6–17.4) (p<0.001). Improvement in prescribing safety was mainly a result of reducing inappropriate abbreviation errors. However, rates for non-abbreviation prescribing errors were significantly higher at 12 weeks post-implementation than at baseline (17.7 per 100 prescriptions, 95% CI 9.5–33.0 versus 8.5 per 100 prescriptions, 95% CI 4.6-15.9) (p <0.001) and no different at baseline than one year (10.2 per 100 prescriptions, 95% CI 6.2–18.6) (p=0.337). Survey results complemented quantitative findings.” \| \| Abramson, 2013^6^  Homegrown  Epic  April 2008 \| Weill Medical College of Cornell University, academically-affiliated, hosptial-based adult internal medicine ambulatory practice in New York City (USA)  Providers (n=16); Prescriptions (n=920)  Mixed methods—one time after deployment:  April to June 2010 \| “We analyzed 1905 prescriptions. The overall prescribing error rate was 3.8 per 100 prescriptions (95% CI 2.8 to 5.1). Error rates were significantly lower 2 years after transition (p<0.001 compared to preimplementation, 12 weeks and 1 year after transition). Rates of near misses remained unchanged. Providers positively appreciated most system refinements, particularly reduced alert firing.” \| \| Barnett, 2016^1^  Various  Not reported  2011 to 2012 \| hospitals that implemented a new inpatient EHR in 2011-21 with a single verifiable "go live" date" (USA)  Hospitals (n=416)  Time series:  2011 to 2012 \| “Before and after implementation, characteristics of admissions were similar in both study and control hospitals. Among study hospitals, unadjusted 30 day mortality (6.74% to 7.15%, P=0.06) and adverse safety event rates (10.5 to 11.4 events per 1000 admissions, P=0.34) did not significantly change after implementation of EHRs. There was an unadjusted decrease in 30 day readmission rates, from 19.9% to 19.0% post-implementation (P=0.02). In difference-indifferences analysis, however, there was no significant change in any outcome between pre-implementation and post-implementation periods (all P≥0.13).” \| \| Colicchio, 2018^3^  Homegrown  Cerner  February 2015 to October 2016 \| Intermountain Healthcare, a no-for-profit, integrated care delivery system of 22 hospitals and over 185 clinics covering Utah and southern Idaho (USA)  Hospitals (n=6) and Primary care clinics (n=49) in 5 regions  Time series:  February 2013 to July 2017 \| “The proposed methodology was successfully implemented and significant changes were observed in most measured variables. A significant change attributable to the intervention was observed in 12 (29%) measures in three or more regions; in 32 (78%) measures in two or more regions; and in 40 (98%) measures in at least one region. A similar pattern (i.e., same impact in three or more regions) was detected for nine (22%) measures, a mixed pattern (i.e., same impact in two regions, and different impact in other regions) was detected for nine (22%) measures, and an inconsistent pattern (i.e., did not detect the same impact across regions) was detected for 23 (56%) measures.” \| \| Friebe, 2020^7^  RxStar  Epic  November 2017 \| Vanderbilt University Medical Center (USA)  Prescriptions (n=359,393)  Time series:  July 2014 to December 2019 \| “Prescribing rates of PIMs decreased 5.2% (13.5 per 100 prescriptions to 12.8 per 100 prescriptions; p < 0.0001) corresponding to the implementation of alternatives CDS in the legacy EHR. After migration of the alternative CDS from the legacy to the new EHR system, PIM prescribing rates dropped an additional 18.8% (10.4 per 100 prescriptions; p < 0.0001). Acceptance rates of the alternative recommendations for PIMs was low overall at 11.1%.” \| \| Whalen, 2018^8^  Homegrown  Epic  April 2016 \| Massachusetts General Hospital, a 999-bed medical center with 114 pediatric beds, pediatric emergency department, pediatric subspecialty care, pediatric intensive care, and neonatal intensive care. (USA)  Not reported  Mixed methods—Time series; qualitative analysis of errors:  October 2015 to August 2016 \| “After implementation, there was a 5-fold increase in the overall number of medication safety reports; by the third month the rate of reported medication errors had returned to baseline. The majority of reports were near misses. Three major safety themes arose: (1) enterprise logic in rounding of doses and dosing volumes; (2) ordering clinician seeing a concentration and product when ordering medications; and (3) the need for standardized dosing units through age contexts created issues with continuous infusions and pump library safeguards.” \| \| Wright, 2018^9^  Homegrown  Epic  May 2015 \| Brigham and Women's Hospital (USA)  Not reported  Time series:  November 2014 to November 2015 \| “Overall interruptive DDI alert burden increased by a factor of 6 from the legacy EHR to the commercial EHR. The acceptance rate for the most severe alerts fell from 100 to 8.4%, and from 29.3 to 7.5% for medium severity alerts (P < 0.001). After disabling the least severe alerts, total DDI alert burden fell by 50.5%, and acceptance of Tier 1 alerts rose from 9.1 to 12.7% (P < 0.01).” \| \| **CLINICAL CARE: *Workflow/Productivity* (n=7)** \| \| \| \| **Author, year**  **Previous EHR**  **New HER**  **Timing of transition** \| **Setting (Country)**  **Sample size (response rate)**  **Study design: Timing of data collection** \| **Findings from abstract** \| \| Calder-Sprackman, 2021^10^  Homegrown  Epic  June 2019 \| Ottawa Hospital, an acadmic tertiary care hospital with 2 emergency department campuses, ~174,000 patient visits per year (Canada)  Physicians (n=15, 100%)  Quantitative, before and two times after deployment:  March 2019 to March 2020 \| “Physicians saw fewer patients per shift during go-live (0.51 patient/hour, P < 0.01), patient efficiency increased in post-implementation but did not recover to baseline (−0.31 patient/hour, P = 0.03). From pre-implementation to post-implementation, we observed a trend towards increased physician time spent charting (+54 seconds/patient, P = 0.05) and documenting (+36 seconds/patient, P = 0.36); time spent doing direct patient care trended towards decreasing (−0.43 seconds/patient, P =0.23). A small percentage of shifts were spent receiving technical support and time spent on teaching activities remained relatively stable during EHR transition.” \| \| Colicchio, 2018^3^  Homegrown  Cerner  February 2015 to October 2016 \| Intermountain Healthcare, a no-for-profit, integrated care delivery system of 22 hospitals and over 185 clinics covering Utah and southern Idaho (USA)  Hospitals (n=6) and Primary care clinics (n=49) in 5 regions  Time series:  February 2013 to July 2017 \| “The proposed methodology was successfully implemented and significant changes were observed in most measured variables. A significant change attributable to the intervention was observed in 12 (29%) measures in three or more regions; in 32 (78%) measures in two or more regions; and in 40 (98%) measures in at least one region. A similar pattern (i.e., same impact in three or more regions) was detected for nine (22%) measures, a mixed pattern (i.e., same impact in two regions, and different impact in other regions) was detected for nine (22%) measures, and an inconsistent pattern (i.e., did not detect the same impact across regions) was detected for 23 (56%) measures.” \| \| Dunn Lopez, 2021^11^  Homegrown  Not reported  Not reported \| 2 ambulatory urgent care centers within one healthcare system using the same EHR system in a small Midwestern metropolitan area (USA)  Nursing staff (n=22) and Provider staff (n=22), (40%)  Mixed methods: quantitative, before and two times after deployment; heuristic evaluation:  2 weeks prior to new EHR implementation, 6-8 months post, and 30-32 months post \| “We found significant increase in perceived workload post-implementation that persisted for 2.5 years (p < .001). The workload increase was associated with usability ratings, which in turn may relate to EHR interface design violations identified by a heuristic evaluation. Our findings suggest further innovation and attention to interface design flaws are needed to improve EHR usability and reduce clinician workload.” \| \| Pandit, 2013^12^  Homegrown  Allscripts  Not reported \| Glaucoma Service at the Wilmer Eye Institute of the Johns Hopkins University School of Medicine (USA)  2 week pre-transition: Detailed Timings (n=44), Patient Surveys (n=44), Clinic Flow (n=94); 2 weeks post-transition: Detailed Timings (n=49), Patient Surveys (n=49) Clinic Flow (n=103); 6 months post-EHR: Detailed Timings (n=38), Patient Surveys (n=38), Clinic Flow (n=76)  Quantitative, before and two times after deployment:  2 weeks pre-transition, 2 weeks post-transition, 6 months post \| “Two weeks after the transition, physicians spent more time with patients overall compared with baseline (8.4 vs. 11.6 minutes), reviewing paper records (0.2 vs. 0.6 minutes), and on computer-related tasks (2.3 vs. 4.2 minutes). At 6 months after EHR transition, physicians also spent more time compared with baseline on the clinical examination (5.1 vs. 6.4 minutes). There was a relative decrease in the percentage of patient time spent waiting to see the physician, although patients’ perceptions of their visit and the EHR remained largely unchanged. Annual clinic volumes also were unaffected by the new EHR.” \| \| Reeves, 2020^13^  Homegrown  Epic  August 2019 \| UCSD Student Health & Well-being, offering campus-based health services to over 39,000 erolled students (USA)  Not reported  Quantitative, before and one time after deployment:  November 2018 to August 2019 \| “36,023 student-patient medical records were created. EHR-integration increased security while creating visibility to 19,700 shared patient visits and records from 236 health systems across the country over 6months. Benefits for the COVID-19 response included access to screening tools, decision support, telehealth, patient alerting system, reporting and analytics, COVID-19 dashboard, and increased testing capabilities.” \| \| Tan, 2017^14^  Sunquest  Beaker CP  February 2015 \| Stanford University Medical Center, comprises of Stanford Health System (613-bed adult hospital and clinics) and Stanford Children's Health (311-bed Lucile Packard Children's Hopsital and clinics) (USA)  Not reported  Time series:  October 2016 to February 2016 \| “Test turnaround times showed improvement from historical baselines, mostly because of the implementation of [positive patient identification] PPID. PPID also resulted in significant reduction in mislabeled specimens.” \| \| Zheng, 2020^15^  Not reported  Not reported  June 2018 \| Mayo Clinic Hopsital (Rochester) in Minnesota (USA)  Video data from 10 cases capturing nurse's pre-op workflow: pre (n=5), post (n=5)  Quantitative, before and one time after deployment:  2016; 2018 \| “The results showed that the EHR conversion brought a significant decrease in the patient case time and a reduced percentage of time using EHR. PreOp nurses spent a higher proportion of time caring for the patient, while the important tasks were completed in a more continuous pattern after the EHR conversion. The workflow variance was due to different nurse’s cognitive process and the task time change was reduced because of some new interface features in the new EHR systems.” \|   **PROVIDER PERSPECTIVES (n=11)** | | |
| **Author, year**  **Previous EHR**  **New HER**  **Timing of transition** | **Setting (Country)**  **Sample size (response rate)**  **Study design: Timing of data collection** | **Findings from abstract** |
| Abramson, 2012^16^  Homegrown  Not reported  April 2008 | Weill Medical College of Cornell University, academically-affiliated, hosptial-based adult internal medicine ambulatory practice in New York City (USA)  Physicians (n=16)  Cross-sectional (qualitative, one time after deployment):  January to November 2009 | “We identified key themes describing physician experiences. Despite intensive effort by the information systems team to ease the transition, even these experienced eprescribers found transitioning extremely difficult. The commercial EHR was not perceived as improving medication safety, despite having more robust CDS. Additionally, physicians felt the commercial EHR was too complex, reducing their efficiency.” |
| Abramson, 2016^17^  Homegrown  Epic  April 2008 | Weill Medical College of Cornell University, academically-affiliated, hosptial-based adult internal medicine ambulatory practice in New York City (USA)  Internists (n=13, 87%)  Cross-sectional (qualitative, one time after deployment):  2012 | “We identified five themes: efficiency and usability, effects on safety, ongoing training requirements, customization, and competing priorities for the EHR. We found that for even experienced e-prescribers, achieving prior levels of perceived prescribing efficiency took nearly two years. Despite the fact that speed in performing prescribing-related tasks was highly important, most were still not utilizing system short cuts or customization features designed to maximize efficiency. Alert fatigue remained common. However, direct transmission of prescriptions to pharmacies was highly valued and its benefits generally outweighed the other features considered poorly designed for physician workflow.” |
| Adler, 2015^18^  Various  Various  2010 to July 2014 | nationwide survey of family physicians: 50% worked in practices owned by a hospital, health system, university; 41% in physician owned practice; 4 percent in fedetally qualified health centers (USA)  Physicians (n=305)  Cross-sectional (quantitative, one time after deployment):  July to September 2014 | “Changing EHR systems is a major endeavor that typically requires large time investments from both users and management and occasions lost productivity, the challenge of learning a new EHR, and significant costs. It can also mean a step backwards, at least temporarily, if significant amounts of data cannot be converted electronically...Does making a switch improve things? It does appear to help add useful functionality and to help users achieve meaningful use. It doesn’t, however, appear to make them happier or to lead consistently to better overall EHR performance. Users were about equally split on whether they had better usability and speed, lower costs, and fewer technical glitches with their new EHR than with their old one. They were also equally split on whether they were happy with the new EHR. Disturbingly, more users disagreed that their new EHR improved productivity than agreed (49 percent vs. 28 percent). One could attribute this to the learning curve of a new EHR, but something else may be going on, because 62 percent of respondents had made their switch more than a year earlier.” |
| Hanauer, 2017^19^  Homegrown  Epic  August 2012 | University of Michigan Health System (USA)  -1 month (n=174, 33%), +3 months (n=236, 44%), +6 months (n=193, 36%), +13 months (n=203, 39%), +25 months (n=179, 35%)  Time series:  July 2012 to September 2014 | “A J-curve was not found for any measures, including workflow, safety, communication, and satisfaction. Only the reminders and alerts measure dropped and then returned to baseline (U-curve); a few remained flatlined. Most dropped and remained below baseline (L-curve). The only measure that remained above baseline was documenting in the exam room with the patient.” |
| Krousel-Wood, 2017^20^  Ochsner  Epic  2012 to 2013 | a large, nonprofit, multispecialty academic health care system with 8 hospitals and 38 clinics located in the urban and rural regions of southeastern Louisiana (USA)  Providers (n=233, 30%)  Quantitative, before and two times after deployment:  Q1 2011 to Q1 2015 | “A total of 223 providers completed all 3 surveys (30% response rate): 85.6% had outpatient practices, 56.5% were >45 years old, and 23.8% were primary care providers. The percentage of providers with positive perceptions significantly increased from baseline to long-term follow-up for patient communication, hospital transitions – access to clinical information, preventive care delivery, preventive care prompt, preventive lab prompt, satisfaction with system reliability, and sharing medical information (P<.05 for each). The percentage of providers with positive perceptions significantly decreased over time for overall satisfaction, productivity, better patient care, clinical decision quality, easy access to patient information, monitoring patients, more time for patients, coordination of care, computer access, adequate resources, and satisfaction with ease of use (P<0.05 for each). Results varied by subgroup.” |
| Pantaleoni, 2015^21^  Not reported  Not reported  Not reported | Stanford Children's Health, which includes Lucile Packard Children's Hospital and ~100 network physicians in a medical foundation (USA)  Providers (n=1,013)  Cross-sectional (quantitative, one time after deployment):  8 weeks prior to go-live | “Evaluations of classroom training, obtained at the conclusion of each class, revealed high physician satisfaction with the program. Free-text comments from learners focused on duration and timing of training, the learning environment, quality of the instructors, and specificity of training to their role or department. Based upon participant feedback and institutional experience, best practice recommendations, including physician engagement, curricular design, and assessment of proficiency and recognition, are suggested for future provider EMR training programs.” |
| Pfoh, 2012^22^  Homegrown  Not reported  April 2008 | 6 academic, urban ambulatory medical practices in NYC on two separate campuses (USA)  Providers (n=197, 64%)  Cross-sectional (quantitative, one time after deployment):  February 2008 to November 2008 | “The response rate was 64% (n = 197).Asmall majority were satisfied with the new EHR (64%, n = 120). Providers who reported satisfaction with overall quality of work life, their workload and the transition were more likely to be satisfied with the new HER (P < 0.01). Providers who reported using the Internet at least daily were also more likely to be satisfied with the new EHR (P < 0.05). In a multivariate model, satisfaction with the transition was a strong predictor of satisfaction with the new EHR (P < 0.01). Barriers to satisfaction include dissatisfaction with: maintaining problem and medication lists, tracking health maintenance information, referring to clinical practice guidelines and ordering laboratory and radiology tests (P < 0.01).” |
| Pirtle, 2019^23^  Homegrown  Epic  November 2017 | Vanderbilt Univeristy Medical Center (USA)  Physicians: Pre (n=37), Post go-live (n=27)  Mixed methods—quantitative, before and one time after deployment; qualitative, one time after deployment:  October 2017; February 2018 to March 2018 | “Themes that emerged from the interviews included stress and anxiety, the desire for more realistic training environments tailored to specialty needs, and concerns about the duration of time between training and implementation.” |
| Zandieh, 2012^24^  Various  Various  April 2008 | 6 academic-affiliated ambulatory care practices in New York City (USA)  Ambulatory care practitioners: Pre=162 (75%), Post=197 (64%)  Quantitative, before and one time after deployment:  March to June 2006; February to November 2008 | “523 subjects were eligible: 217 were available before transition and 306 after transition. 162 pre-transition and 197 post-transition responses were received, yielding 75% and 64% response rates, respectively. Practitioners were more satisfied with the newer EHRs (64%) compared with the older (56%) (p=0.15) and a small majority (58%) were satisfied with the transition. Practitioners’ satisfaction with the older EHRs for completing clinical tasks was high. The newer EHRs exceeded practitioner expectations regarding remote access (61% vs 74%; p=0.03). However, the newer EHRs did not meet practitioners’ expectations regarding their ability to perform clinical tasks, or more globally, improve medication safety (81% vs 61%; p<0.001), efficiency (70% vs 44%; p<0.001), and quality of care (77% vs 67%; p=0.04).” |
| **PATIENT SATISFACTION (n=4)** | |  |
| **Author, year**  **Previous EHR**  **New HER**  **Timing of transition** | **Setting (Country)**  **Sample size (response rate)**  **Study design: Timing of data collection** | **Findings from abstract** |
| Monturo, 2022^25^  Other  Epic  Not reported | 248-bed not-for-profit community hospital in Pennsylvania (USA)  165 patients (random sample of n=55 at 3 different time points)  Quantitative, before and two times after deployment:  baseline (before EHR change), 6 weeks post-implementation, and 6 months post-implementation | “The transition in the EHR did not seem to affect the overall patient experience [related to point-of-care computer use, user’s comfort, or impact on patient relationship with a nurse or provider].” |
| North, 2020^26^  Various  Epic  July 2017 to Oct 2018 | 6 clinic practices in Wisconsin, Minnesota, Arizona, Florida within the Mayo Clinic Health System (USA)  Not reported  Time series:  July 2016 to January 2020 | Significant drops in patient satisfaction were associated with the EHR switch. Patient satisfaction with access (ease of getting clinic on phone, ease of scheduling appointments, etc.) was most affected (range of 6 sites absolute decline: -3.4% to -8.8%; all significant at 99% confidence interval). Satisfaction with providers was least affected (range of 6 sites absolute decline: -0.5% to -2.8%; 4 of 6 sites significant at 99% confidence interval). After 9-15 months, patient satisfaction with access climbed back to pre-EHR switch levels. |
| Pandit, 2013^12^  Homegrown  Allscripts  Not reported | Glaucoma Service at the Wilmer Eye Institute of the Johns Hopkins University School of Medicine (USA)  2 week pre-transition: Detailed Timings (n=44), Patient Surveys (n=44), Clinic Flow (n=94); 2 weeks post-transition: Detailed Timings (n=49), Patient Surveys (n=49) Clinic Flow (n=103); 6 months post-EHR: Detailed Timings (n=38), Patient Surveys (n=38), Clinic Flow (n=76)  Quantitative, before and two times after deployment:  2 weeks pre-transition, 2 weeks post-transition, 6 months post | “Two weeks after the transition, physicians spent more time with patients overall compared with baseline (8.4 vs. 11.6 minutes), reviewing paper records (0.2 vs. 0.6 minutes), and on computer-related tasks (2.3 vs. 4.2 minutes). At 6 months after EHR transition, physicians also spent more time compared with baseline on the clinical examination (5.1 vs. 6.4 minutes). There was a relative decrease in the percentage of patient time spent waiting to see the physician, although patients’ perceptions of their visit and the EHR remained largely unchanged. Annual clinic volumes also were unaffected by the new EHR.” |
| Tian, 2021^27^  Various  Epic  Oct 2017-June 2018 | 10 adult hospitals from a midwest healthcare system  34,425 patients (across the study period)  Time series:  July 2015-March 2019 (pre-implementation and quarterly post-implementation) | “No statistically significant changes were observed [on the Hospital Consumer Assessment of Healthcare Providers and Systems results] in the transition, second, or third quarters post-implementation. Patient experience scores returned to baseline level after two quarters and the impact from EHR transition appeared to be temporary.” |
| **DATA MIGRATION (n=8)** | |  |
| **Author, year**  **Previous EHR**  **New HER**  **Timing of transition** | **Setting (Country)**  **Sample size (response rate)**  **Study design: Timing of data collection** | **Findings from abstract** |
| Behlen, 2000^35^  Homegrown  QuadRIS  1999 | University of Chicago Department of Radiology (USA)  Not reported  Quantitative, one time after deployment:  Not reported | We find that for two PACS archives, both organized according to the Digital Imaging and Communications in Medicine (DICOM) information model, data may be transferred with full fidelity, but the time required for transfer is significant. Transfer from off-line backup media was found to be faster than transfer from our robotic tape library. In contrast, the RIS replacement required extensive labor to translate prior data between dissimilar information models, and some data were inevitably Iost in the translation. Standards for RIS information models are needed to promote the migration of data without loss of content. |
| Epstein, 2019^28^  Various  Epic  April 2017 | 3 hospitals that are part of the associated medical center (Thomas Jefferson University Hospital, the Jefferson Hospital for Neuroscience, the Jefferson Surgical Center) Philadelphia, PA (USA)  Not reported  Time series:  January 2011 to May 2017 | “At the time of implementation of the EHR, in 44.8% (SE = 0.3%) of cases, there was a prior anesthetic record for the patient that had been documented in the legacy AIMS. Following EHR implementation, the mean number of preoperative clinical views of all prior anesthetic records divided by the total number of cases performed decreased to 2.3% (0.3%) from the baseline of 25.1%(0.8%). The estimated ratio of the 2 means was 0.18 (95% CI 0.11 to 0.31, P < 0.00001). For views of unique records, the decrease was to 2.2% (0.3%) from the baseline of 18.3% (0.5%). The estimated ratio was 0.23 (95% CI 0.15 to 0.35, P < 0.00001). These results show that, following conversion to an integrated EHR, providing access to historical anesthesia records by maintaining the legacy AIMSis not an effective strategy to promote review of such records as part of the preoperative evaluation process. Because such records provide important information for many patients, providing linked access to such records within the EHR as part of the patient encounter may be a more effective approach.” |
| MacKenzie, 2021^29^  Not reported  Not reported  Not reported | 602-bed urban tertiary care hospital with 20 ambulatory care clinics that are part of a larger health-network (USA)  Physicians (n=17, 90%)  Cross-sectional (quantitative, one time after deployment):  Not reported | “Detailed data migration feedback was obtained from 90% of participants. Depending on the specialty, requests for historical laboratory values ranged from 2 to as many as 145 unique laboratory types. Lookback periods requested by physicians varied and were ultimately assigned to provide the most clinical data. This clinical information was then combined to synthesize an overall proposed data migration request on behalf of the institution.” |
| Makar, 2014^30^  Various  COSMIC, TajeCare  Stockholm 2000 to 2007; Uppsala 2003 to 2008; Ostergotland 2005 to 2008 | 3 counties in Sweden (Stockholm, Uppsale, Ostergotland) (Sweden)  Project leaders & team leaders for interviews (n=7); Healt care personnel for questionnaires (n=53)  Mixed methods—one time after deployment:  Interviews = March to April 2014; Questionnaire = April 2014 | “Using the legacy systems and the new ones under a certain period, accessing the information in the legacy systems via a link (to a database) that connects the new system with the data exported from the legacy system and manually entering the data to the new system were the three main identified approaches that had been used. Automatic data transfer is currently infeasible given the architectural differences between the systems. Although relatively long time has passed since the transitions took place, 75% of the users who responded to the questionnaire are still dependent on the information from the legacy systems and 60% of the users expressed having no or very slight influences on the decisions made regarding the legacy information.” |
| Michel, 2014^31^  Not reported  Not reported  2013 | ~1000-bed tertiary academic medical center with spectrum of ambulatory clinics (USA)  Immunization data points (n=502,095)  Cross-sectional (quantitative, one time after deployment):  Not reported | “We completed the data migration from two facilities in 16.8 hours with no data loss or corruption. We successfully populated the future EMR with 99.16% of our legacy immunization data – 500,906 records – just prior to our EMR transition date. A subset of immunizations, first recognized during clinical care, had not originally been extracted from the legacy systems. Once identified, this data – 1,695 records – was migrated using the same process with minimal additional effort.” |
| Pageler, 2016^32^  Cerner  Epic  May 2014 | Lucile Packard Children’s Hospital Stanford (LPCHS), a 303 bed, freestanding, quaternary care, academic children’s hospital (USA)  15% of total records (>300,000 patient records)  Cross-sectional (quantitative, one time after deployment):  Not reported | “The only error identified in the post go-live period was a failure to migrate some clinical notes, which was uNot reportedelated to the validation process. No errors in the migrated data were found during the 12- month post-implementation period.” |
| Schreiber, 2020^33^  Various  Epic  2015 (Holy Spirit), 2007 (Reliant) | Geisinger Holy Spirit, independent not-for-profit community hospital with 270 acute inpatient beds; Reliant Medical Group, a 500-provider multispecialty group practice in Central and MetroWest Massachusetts (USA)  Health system (n=2)  Systematic Review:  N/A | “The two new case studies contrast starkly: one relied on manual abstraction and data entry, whereas the other leveraged several electronic tools. The literature reflects this diversity of approach: no two sites have reported the same approaches. The authors identify nine domains of potential consequences of the currently available techniques and offer mitigating strategies.” |
| Wang, 2020^34^  Not reported  Not reported  March 2018 | Northwestern Memorial Hospital (USA)  Not reported  Cross-sectional (quantitative, one time after deployment):  June 2002 to November 2015 | “The review, mapping, and migration revealed interesting issues and challenges with the free-text allergy information and the mapping in preparation for implementation in the new EHR system. These findings provide insights that can form the basis of guidelines for future mapping and migration efforts involving free-text allergy data. As part of this process, we generate and make freely available AllergyMap, a mapping between free-text entered allergy medication to standard non-proprietary ontologies.” |
| **OTHER TOPICS (n=5)** | |  |
| **Author, year**  **Previous EHR**  **New HER**  **Timing of transition** | **Setting (Country)**  **Sample size (response rate)**  **Study design: Timing of data collection** | **Findings from abstract** |
| Amlung, 2020^35^  Various  various  Various | Various healthcare organizations that had transitioned to a new EHR system between 2009 to 2019 (USA)  Healthcare organizations (n=13)  Mixed methods—one time after deployment:  May to July 2019 | “The interviewees emphasized the importance of organizational and process revision during modernization, converting historical data, and clinical and leadership involvement. HIT implementation required technological and infrastructure redesign, additional training, and workflow reconfiguration. Motivations for modernization included EHR usability dissatisfaction, revenue enhancements, and improved clinical operations. Decision-making strategies, primarily during HIT selection, included meetings with stakeholders. Successful modernization resulted in improvements in clinical operations, patient experience, and financial outlay.” |
| Lammers, 2011^36^  Various  Various  2003 to 2008 | large national sample of non-federal acute care hospitals (USA)  2003 (n=2,136), 2008 (n=3,677)  Cross-sectional (quantitative, one time after deployment):  2003; 2008 | “We find that there has been considerable switching between vendors by hospitals, including some hospitals switching away from automated systems all together. Furthermore, our descriptive cross-sectional analysis reveals various hospital characteristics which are associated with vendor switching and dropping, including lower constraints on hospitals’ financial resources, nonprofit ownership, and having some form of integrated arrangement with physicians.” |
| McEvoy, 2018^37^  various  Various  2011 to 2015 | hospitals that implemented new EHR between 2011 and 2015 (USA)  Hospitals (n=32)  Quantitative, before and one time after deployment:  2011 to 2016 | “After implementing an EHR, 7 hospitals had a bond downgrade, 7 had a bond upgrade, and 18 had no changes. There was no difference in the likelihood of bond rating changes or in changes to NISP following HER go-live when compared to control hospitals.” |
| Umstead, 2021^38^  Homegrown  Not reported  November 2017 | Vanderbilt Unviersity Medical Center, a large-scale healthcare institution with 18,000+ employees, 4 hospitals, and 1100+ licensed beds (USA)  Clinical sites (n=12)  Cross-sectional (qualitative, during transition):  November 2017 to December 2018 | “We found that support personnel possessing both contextual knowledge of the institution’s workflow and training in the new technology were the most successful in mediation of adoption and use. Those that lacked context of either technology or institutional workflow often displayed barriers in communication, trust, and active problem solving.” |
| Yuan, 2021^39^  Not reported  Not reported  2012 to 2015 | US-based healthcare providers classified as rural hospitals, community health centers, small practice, public hospitals, underserved settings, practice consortia, and rural clinics, part of the Office of National Coordinator for Health Information Technol (USA)  Not reported  Time series:  2012 to 2015 | “With the data from the Office of National Coordinator for Health Information Technology—Regional Extension Centers Program, this study finds that healthcare providers have achieved progress in the EHR conversion at both the primary and advanced stage. However, the levels of progress made at different stages of EHR conversion vary for different providers. For rural and underserved healthcare settings, the progress made at the advanced stage is smaller than that at the primary stage, contrary to the case for other kinds of providers. Moreover, although the greater progress has been made at the advanced stage for some kinds of providers, the overall level of EHR conversion for various healthcare providers is far greater at the primary stage than at the advanced stage.” |

**References for Evidence Tables**

1. Barnett ML, Mehrotra, A., & Jena, A. B. Adverse inpatient outcomes during the transition to a new electronic health record system: observational study. *BMJ*. 2016;354(i3835)

2. Binney G C-PT, Roomian T, et al. Effect of an electronic health record transition on the provision of recommended well child services in pediatric primary care practices. *Clin Pediatr (Phila)* 2020;59(2):188–197.

3. Colicchio TK, Del Fiol G, Scammon DL, Facelli JC, Bowes WA, 3rd, Narus SP. Comprehensive methodology to monitor longitudinal change patterns during EHR implementations: a case study at a large health care delivery network. *J Biomed Inform*. Jul 2018;83:40-53. doi:10.1016/j.jbi.2018.05.018

4. Sivashanker K, Bell G, Khorasani R, et al. Electronic Health Record Transition and Impact on Screening Test Follow-Up. *Jt Comm J Qual Patient Saf*. Mar 31 2021;doi:10.1016/j.jcjq.2021.03.010

5. Abramson EL, Malhotra S, Fischer K, et al. Transitioning between electronic health records: effects on ambulatory prescribing safety. *J Gen Intern Med*. Aug 2011;26(8):868-74. doi:10.1007/s11606-011-1703-z

6. Abramson EL, Malhotra S, Osorio SN, et al. A long-term follow-up evaluation of electronic health record prescribing safety. *J Am Med Inform Assoc*. Jun 2013;20(e1):e52-8. doi:10.1136/amiajnl-2012-001328

7. Friebe MP, LeGrand JR, Shepherd BE, Breeden EA, Nelson SD. Reducing Inappropriate Outpatient Medication Prescribing in Older Adults across Electronic Health Record Systems. *Appl Clin Inform*. Oct 2020;11(5):865-872. doi:10.1055/s-0040-1721398

8. Whalen K, Lynch E, Moawad I, John T, Lozowski D, Cummings BM. Transition to a new electronic health record and pediatric medication safety: lessons learned in pediatrics within a large academic health system. *J Am Med Inform Assoc*. Jul 1 2018;25(7):848-854. doi:10.1093/jamia/ocy034

9. Wright A, Aaron S, Seger DL, Samal L, Schiff GD, Bates DW. Reduced Effectiveness of Interruptive Drug-Drug Interaction Alerts after Conversion to a Commercial Electronic Health Record. *J Gen Intern Med*. Nov 2018;33(11):1868-1876. doi:10.1007/s11606-018-4415-9

10. Calder-Sprackman S, Clapham G, Kandiah T, et al. The impact of adoption of an electronic health record on emergency physician work: A time motion study. *J Am Coll Emerg Physicians Open*. Feb 2021;2(1):e12362. doi:10.1002/emp2.12362

11. Dunn Lopez K, Chin CL, Leitão Azevedo RF, et al. Electronic health record usability and workload changes over time for provider and nursing staff following transition to new EHR. *Appl Ergon*. May 2021;93:103359. doi:10.1016/j.apergo.2021.103359

12. Pandit RR, Boland MV. The impact of an electronic health record transition on a glaucoma subspecialty practice. *Ophthalmology*. Apr 2013;120(4):753-60. doi:10.1016/j.ophtha.2012.10.002

13. Reeves JJ, Longhurst CA, San Miguel SJ, et al. Bringing student health and Well-Being onto a health system EHR: the benefits of integration in the COVID-19 era. *J Am Coll Health*. Nov 12 2020:1-7. doi:10.1080/07448481.2020.1843468

14. Koppel RL, C. U. Implications of an emerging EHR monoculture for hospitals and healthcare systems. *Journal of the American Medical Informatics Association*. 2014;22(2):465-471.

15. Zheng L, Duncan BJ, Kaufman DR, et al. EHR Conversion on the PreOp Care: A Pre-Post Workflow Comparison. *AMIA Annu Symp Proc*. 2020;2020:1402-1411.

16. Abramson EL, Patel V, Malhotra S, et al. Physician experiences transitioning between an older versus newer electronic health record for electronic prescribing. *Int J Med Inform*. Aug 2012;81(8):539-48. doi:10.1016/j.ijmedinf.2012.02.010

17. Abramson EL, Patel V, Pfoh ER, Kaushal R. How Physician Perspectives on E-Prescribing Evolve over Time. A Case Study Following the Transition between EHRs in an Outpatient Clinic. *Appl Clin Inform*. Oct 26 2016;7(4):994-1006. doi:10.4338/ACI-2016-04-RA-0069

18. Adler KG, Edsall RL. EHR Switch Survey: Responses From 305 Family Physicians. *Fam Pract Manag*. Jan-Feb 2015;22(1):13-8.

19. Hanauer DA, Branford GL, Greenberg G, et al. Two-year longitudinal assessment of physicians' perceptions after replacement of a longstanding homegrown electronic health record: does a J-curve of satisfaction really exist? *J Am Med Inform Assoc*. Apr 1 2017;24(e1):e157-e165. doi:10.1093/jamia/ocw077

20. Krousel-Wood M, McCoy, A. B., Ahia, C., Holt, E. W., Trapani, D. N., Luo, Q., ... & Milani, R. V. . Implementing electronic health records (EHRs): health care provider perceptions before and after transition from a local basic EHR to a commercial comprehensive EHR. . 2017, In Press;Journal of the American Medical Informatics Association.

21. Pantaleoni JL, Stevens LA, Mailes ES, Goad BA, Longhurst CA. Successful physician training program for large scale EMR implementation. *Appl Clin Inform*. 2015;6(1):80-95. doi:10.4338/ACI-2014-09-CR-0076

22. Pfoh ER, Abramson E, Zandieh S, Edwards A, Kaushal R. Satisfaction after the transition between electronic health record systems at six ambulatory practices. *J Eval Clin Pract*. Dec 2012;18(6):1133-9. doi:10.1111/j.1365-2753.2011.01756.x

23. Pirtle CJ, Reeder RR, Lehmann CU, Unertl KM, Lorenzi NM. Physician Perspectives on Training for an EHR Implementation. *Stud Health Technol Inform*. Aug 21 2019;264:1318-1322. doi:10.3233/SHTI190440

24. Zandieh SO, Abramson EL, Pfoh ER, Yoon-Flannery K, Edwards A, Kaushal R. Transitioning between ambulatory EHRs: a study of practitioners' perspectives. *J Am Med Inform Assoc*. May-Jun 2012;19(3):401-6. doi:10.1136/amiajnl-2011-000333

25. Monturo C, Brockway C, Ginev A. Electronic Health Record Transition: The Patient Experience. *Computers, informatics, nursing : CIN*. 2022/01/01/ 2022;40(1):53-60. doi:10.1097/CIN.0000000000000805

26. North F, Pecina JL, Tulledge-Scheitel SM, Chaudhry R, Matulis JC, Ebbert JO. Is a switch to a different electronic health record associated with a change in patient satisfaction? *J Am Med Inform Assoc*. Jun 1 2020;27(6):867-876. doi:10.1093/jamia/ocaa026

27. Tian D, Hoehner CM, Woeltje KF, Luong L, Lane MA. Disrupted and Restored Patient Experience With Transition to New Electronic Health Record System. *Journal of patient experience*. 2021 2021;8:23743735211034064. doi:10.1177/23743735211034064

28. Epstein RH, Dexter F, Schwenk ES. Provider Access to Legacy Electronic Anesthesia Records Following Implementation of an Electronic Health Record System. *J Med Syst*. Mar 16 2019;43(5):105. doi:10.1007/s10916-019-1232-6

29. MacKenzie B, Anaya G, Hu J, Brickman A, Elkin PL, Panesar M. Defining Data Migration Across Multidisciplinary Ambulatory Clinics Using Participatory Design. *Appl Clin Inform*. Mar 2021;12(2):251-258. doi:10.1055/s-0041-1726032

30. Makar M. Dealing with existing data in legacy systems when transitioning between Electronic Health Records in three Swedish counties. . Accessed August 21, 2020, <https://ki.se/sites/default/files/migrate/dealing_mina_makar.pdf>

31. Michel J, Hsiao A, Fenick A. Using a scripted data entry process to transfer legacy immunization data while transitioning between electronic medical record systems. *Appl Clin Inform*. 2014;5(1):284-98. doi:10.4338/ACI-2013-11-RA-0096

32. Pageler NM GGSM, Chandler W, Mailes E, Yang C, Longhurst CA. A rational approach to legacy data validation when transitioning between electronic health record systems. *J Am Med Inform Assoc* 2016;23(5):991–994.

33. Schreiber R, Garber L. Data migration: a thorny issue in electronic health record transitions—case studies and review of the literature. *ACI Open*. 2020;4(01):e48-e58.

34. Wang AY, Osborne JD, Danila MI, Naidech AM, Liebovitz DM. AllergyMap: An Open Source Corpus of Allergy Mention Normalizations. *AMIA Annu Symp Proc*. 2020;2020:1249-1257.

35. Amlung J, Huth H, Cullen T, Sequist T. Modernizing health information technology: lessons from healthcare delivery systems. *JAMIA Open*. Oct 2020;3(3):369-377. doi:10.1093/jamiaopen/ooaa027

36. Lammers EJ, Zheng K. Characteristics associated with hospital health IT vendor switching and dropping. *AMIA Annu Symp Proc*. 2011;2011:742-9.

37. McEvoy D, Barnett ML, Sittig DF, Aaron S, Mehrotra A, Wright A. Changes in hospital bond ratings after the transition to a new electronic health record. *J Am Med Inform Assoc*. May 1 2018;25(5):572-574. doi:10.1093/jamia/ocy007

38. Umstead CN, Unertl KM, Lorenzi NM, Novak LL. Enabling adoption and use of new health information technology during implementation: Roles and strategies for internal and external support personnel. *J Am Med Inform Assoc*. Apr 24 2021;doi:10.1093/jamia/ocab044

39. Yuan B, Li J, Wu P. The effectiveness of electronic health record promotion for healthcare providers in the United States since the Health Information Technology for Economic and Clinical Health Act: An empirical investigation. *Int J Health Plann Manage*. Mar 2021;36(2):334-352. doi:10.1002/hpm.3085
